# The Nucleolar Protein LYAR Facilitates Ribonucleoprotein Assembly of Influenza A Virus

**DOI:** 10.1128/JVI.01042-18

**Published:** 2018-11-12

**Authors:** Cha Yang, Xiaokun Liu, Qingxia Gao, Tailang Cheng, Rong Xiao, Fan Ming, Shishuo Zhang, Meilin Jin, Huanchun Chen, Wenjun Ma, Hongbo Zhou

**Affiliations:** a State Key Laboratory of Agricultural Microbiology, College of Veterinary Medicine, Huazhong Agricultural University, Wuhan, Hubei, People's Republic of China; b Department of Diagnostic Medicine and Pathobiology, Kansas State University, Manhattan, Kansas, USA; c Key Laboratory of Preventive Veterinary Medicine in Hubei Province, the Cooperative Innovation Center for Sustainable Pig Production, Wuhan, Hubei, People's Republic of China; University of Southern California

**Keywords:** influenza A virus, vRNP, host factor, LYAR, viral RNP assembly

## Abstract

Influenza A viral ribonucleoprotein (vRNP) is responsible for transcription and replication of the viral genome in infected cells and depends on host factors for its functions. Identification of the host factors interacting with vRNP not only improves understanding of virus-host interactions but also provides insights into novel mechanisms of viral pathogenicity and the development of new antiviral strategies. Here, we have identified 80 host factors that copurified with vRNP using affinity purification followed by mass spectrometry. LYAR, a cell growth-regulating nucleolar protein, has been shown to be important for influenza A virus replication. During influenza A virus infection, LYAR expression is increased and partly translocates from the nucleolus to the nucleoplasm and cytoplasm. Furthermore, LYAR interacts with RNP subunits, resulting in enhancing viral RNP assembly, thereby facilitating viral RNA synthesis. Taken together, our studies identify a novel vRNP binding host partner important for influenza A virus replication and further reveal the mechanism of LYAR regulating influenza A viral RNA synthesis by facilitating viral RNP assembly.

**IMPORTANCE** Influenza A virus (IAV) must utilize the host cell machinery to replicate, but many of the mechanisms of IAV-host interaction remain poorly understood. Improved understanding of interactions between host factors and vRNP not only increases our basic knowledge of the molecular mechanisms of virus replication and pathogenicity but also provides insights into possible novel antiviral targets that are necessary due to the widespread emergence of drug-resistant IAV strains. Here, we have identified LYAR, a cell growth-regulating nucleolar protein, which interacts with viral RNP components and is important for efficient replication of IAVs and whose role in the IAV life cycle has never been reported. In addition, we further reveal the role of LYAR in viral RNA synthesis. Our results extend and improve current knowledge on the mechanisms of IAV transcription and replication.

## Abstract (precis)

Influenza A virus (IAV) must utilize the host cell machinery to replicate, but many of the mechanisms of IAV-host interaction remain poorly understood. Improved understanding of interactions between host factors and vRNP not only increases our basic knowledge of the molecular mechanisms of virus replication and pathogenicity but also provides insights into possible novel antiviral targets that are necessary due to the widespread emergence of drug-resistant IAV strains. Here, we have identified LYAR, a cell growth-regulating nucleolar protein, which interacts with viral RNP components and is important for efficient replication of IAVs and whose role in the IAV life cycle has never been reported. In addition, we further reveal the role of LYAR in viral RNA synthesis. Our results extend and improve current knowledge on the mechanisms of IAV transcription and replication.

## INTRODUCTION

Influenza A virus (IAV) is an important human pathogen that causes considerable human morbidity and mortality every year (1–3). Although vaccines and antivirals to prevent infection and to treat infected humans are possible, their development is still a significant challenge for public health due to rapid changes of IAVs (4, 5). The emergence of novel and/or drug-resistant IAV strains results in failure of available vaccines and antivirals (6). Therefore, to combat the threats posed by IAVs, a comprehensive understanding of IAV replication and transmission is needed in order to develop effective countermeasures.

Viral RNP (vRNP) plays a central role in virus replication, and each vRNP complex consists of a single-stranded negative-sense genomic RNA, associated with multiple copies of the viral nucleoprotein (NP) and a single trimeric RNA-dependent RNA polymerase complex (RdRp; composed of PB1, PB2, and PA) (7). The polymerase synthesizes all three species RNAs, including viral RNA (vRNA), cRNA, and message RNA (mRNA), using the RNA strand that is encapsidated by the oligomeric NP as a template (8, 9). Within the polymerase complex, polymerase basic protein 1 (PB1) contains the polymerase active site, while polymerase acidic protein (PA) and polymerase basic protein 2 (PB2) initiate the transcription process by cap snatching (10–13). During IAV infection, after the vRNPs are transported into the nucleus, RNA polymerase transcribes the vRNA segments into mRNAs; this process is known as primary transcription. The viral RNA polymerase performs the replication of genomic RNA into a cRNA that serves as a template for more vRNA, and cRNA and vRNA are assembled with newly synthesized viral polymerase and nucleoprotein to form cRNPs and vRNPs, respectively (9). The vRNP assembly is required for the transition from primary transcription to genome replication (14). However, the mechanisms of vRNP assembly and the roles of host proteins in this process are largely unknown. The proposed model for polymerase assembly shows that newly synthesized polymerase subunits are transported into the nucleus in the form of PA-PB1 dimer and PB2 monomer and then assembled into polymerase complex (15–17). However, how the polymerase complexes, NP oligomers, and viral RNAs cooperate to assemble into the final vRNPs remains unknown.

IAV transcription and replication depends on host nuclear machineries, and the interplay between host factors in nucleus and vRNPs is critical for completing this process. During numerous nuclear replicating virus infections, several nuclear domains have been shown to be targeted by viral proteins, such as nucleoli, promyelocytic leukemia (PML) bodies, and nuclear speckles (18–21). The nucleolus is known to be the site of rRNA gene transcription, rRNA processing, and assembly into preribosomal subunits (22, 23), and its nonclassical functions include mRNA transport, sequestration of regulation complex, and regulation of cellular metabolism and cellular stress responses (24). IAV also interacts with the nucleoli to usurp host cell functions and recruits nucleolar proteins to aid its replication in the nucleus; in particular, the vRNP hijacks many nucleolar proteins for its function (25–27). Mayer et al. identified 45 cellular proteins interacting with vRNP or polymerase (28); 11 of them are nucleolar proteins, including the two major constitutive proteins of the nucleolus, nucleolin (NCL or C23) and nucleophosmin (NPM1 or B23), and ribosomal proteins, some of which have also been identified by others using similar proteomic approaches or other experiments (29, 30). In addition, the genome-wide RNA interference (RNAi)-based screens have highlighted the importance of several of these nucleolar proteins for IAV replication (31–33). However, the functional interplay between vRNP and nucleoli remains largely unknown.

Identification of the host proteins that associate with vRNP and the study of their interactions not only improves insights into the molecular mechanisms of viral transcription and replication but also contributes to a deeper understanding of the cell biology of the nuclear components. So far, numerous host factors that interact with vRNP components have been identified, and some have been shown to play roles in virus replication (9, 34); nonetheless, the roles of very few of these host factors have been investigated in detail. In this study, reconstituted IAV vRNPs were purified by Flag-tagged PB1 from human 293T cells using an affinity purification mass spectrometry (AP-MS) strategy. Liquid chromatography-tandem mass spectrometry (LC-MS/MS) was used to analyze the copurified host proteins. Finally, 80 host factors were identified. This complements previous studies in which proteomics-based virus-host interactome screens and genome-wide RNAi-based screens were used to identify host proteins that participate in viral replication, especially those required for viral transcription and replication (28, 29, 32, 35–39). Furthermore, we show that a novel vRNP interacting factor of 80 identified host factors, the cell growth-regulating nucleolar protein LYAR, promotes IAV replication. LYAR contains two C2HC-type zinc finger DNA-binding motifs and a lysine-rich region containing three nuclear localization signals (NLS) and a coiled-coil domain (40). However, the cellular function of LYAR has been poorly studied. Several studies report that LYAR participates in ribosome processing, regulation of gene expression, and cell growth (41–44), but its role in IAV or other virus life cycles has not been reported. Here, we report that LYAR expression is increased during IAV infection and recruited by vRNP to facilitate its assembly.

## RESULTS

### Identification of LYAR as a putative vRNP interacting partner regulating IAV replication.

**FIG 1:**
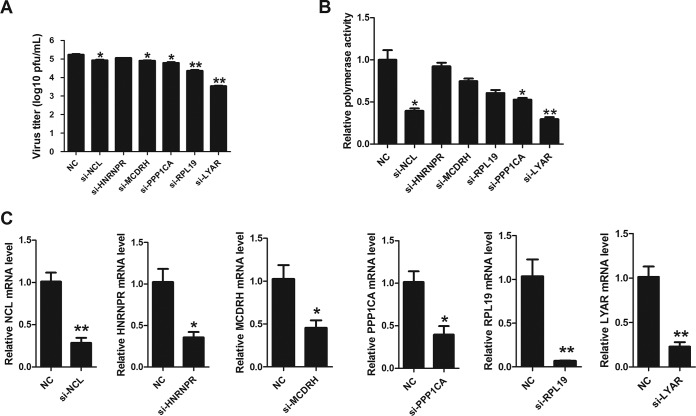
Effects of silenced candidate proteins on IAV replication and polymerase activity. (A) Effects of candidate proteins on virus replication. A549 cells transfected with the indicated siRNAs were infected with the PR8 H1N1 virus (MOI of 0.1) for 24 h, and virus titers were determined by plaque assay on MDCK cells. (B) Effects of candidate proteins on polymerase activity. HEK293T cells were transfected with the indicated siRNAs and viral RNP reconstitution plasmids (pCDNA-3.1-PB1, -PB2, -PA, and -NP, pPolI-Luc, and Renilla); polymerase activity was measured at 24 h posttransfection. (C) The silencing efficiency of the indicated siRNAs was determined by real-time PCR. For all experiments, the data are presented as the means ± SD from three independent experiments (*, *P* < 0.05; **, *P* < 0.01; ***, *P* < 0.001; all by two-tailed Student's *t* test).

As detailed in the supplemental material using the reconstituted IAV vRNP (see Fig. S1 and S2 in the supplemental material), we identified 80 host proteins that copurified with vRNP by using affinity purification followed by mass spectrometry (Fig. S3 and Table S2); 61 of them are identified for the first time as potential vRNP-interacting partners despite their also interacting with other influenza viral proteins, and 21 are claimed to interact with all subunits of the RNP (Fig. S3 and Table S3). For further study, we focused on the nucleolar proteins due to their potential roles in vRNP function and poor knowledge of their interactions with IAV vRNP. Seventeen proteins identified were localized in the nucleolus (Fig. S4 and Table S4), and seven of them were ribosomal proteins (RPL family proteins) that were previously shown to bind viral RNP or RdRp (28, 29). NCL is the major nucleolar protein of growing eukaryotic cells with multiple functions whose roles in IAV replication are well established (26, 45, 46). Of the remaining proteins, only five of them, concerning influenza virus replication, were not determined (Table S5). Therefore, a short interfering RNA (siRNA) screen (LYAR, PPP1CA, HNRNPR, MCDRH, RPL19, and NCL) was performed to investigate their effects on IAV replication and polymerase activity, and NCL was used to serve as the positive control. Results showed that knockdown of LYAR, MCDRH, RPL19, or PPP1CA led to a significant decrease of virus titer at 24 h postinfection (hpi) compared to that of the NC control (Fig. 1A). Noticeably, a greater reduction in both virus titers (approximately 50-fold decrease) and polymerase activity (approximately 60% reduction) was observed in the si-LYAR group than in the NC controls (Fig. 1A and B). These results indicate an important role of LYAR in IAV replication. The siRNA silencing efficiency for tested genes was detected by quantitative reverse transcription-PCR (qRT-PCR), and the mRNA levels of each gene in silencing cells were reduced more than 50% compared to those in negative-control cells (Fig. 1C). Based on these results, we focused on the LYAR-vRNP interaction in our further studies.

### LYAR interacts with IAV RNP subunits.

**FIG 2:**
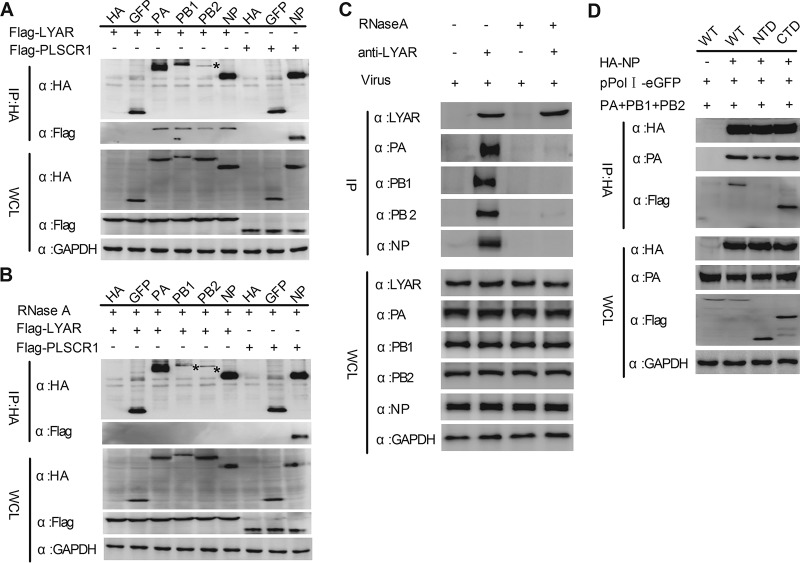
Interactions between LYAR and viral RNP components. (A and B) The interactions between LYAR and RNP components in transfected cells. HEK293T cells transfected with the indicated plasmids were lysed at 24 h posttransfection. The cell lysates were left untreated (A) or were treated with 100 U RNase A (B) at 37°C for 1 h. Co-IP was performed using an anti-HA antibody, followed by Western blotting to detect the viral proteins and LYAR and PLSCR1 by using anti-HA and anti-Flag antibodies, respectively (asterisks indicate specific PB2 or PB1 detected). (C) The interactions between endogenous LYAR and RNP components in IAV-infected cells. A549 cells were infected with the PR8 H1N1 virus (MOI of 2) for 10 h, cells were treated as described above, and Co-IP was performed using an anti-LYAR mouse antibody or mouse IgG. The endogenous LYAR and coprecipitated viral proteins were detected by using an anti-LYAR antibody and the antibodies against individual viral proteins, respectively. Mouse IgG served as the negative control. (D) The interactions between reconstituted vRNP and LYAR or its truncation mutants. HEK293T cells were cotransfected with the vRNP reconstitution plasmids (pCDNA 3.1-PB1, -PB2, and -PA, pPolI-eGFP, HA-NP, or pCAGGS-HA) along with Flag-LYAR (WT), Flag-LYAR N-terminal domain (NTD; amino acids 1 to 167), or Flag-LYAR C-terminal domain (CTD; amino acids 168 to 379). Co-IP was performed using an anti-HA antibody to immunoprecipitate PA and LYAR or its truncation mutants. For all of these experiments, GAPDH served as the loading control.

Interaction between LYAR and each individual component of the RNP was determined. Flag-LYAR and hemagglutinin (HA)-tagged PA, PB1, PB2, and NP, or HA-tagged green fluorescent protein (GFP) and HA (negative controls), were coexpressed in HEK293T cells, and a coimmunoprecipitation (Co-IP) assay was performed using an anti-HA tag monoclonal antibody. Results showed that LYAR was coprecipitated by PA, PB1, PB2, and NP but not the negative controls GFP and HA, suggesting that LYAR specifically interacts with all of the components of RNP (Fig. 2A). Since LYAR and all of the RNP components are RNA binding proteins, we hypothesized that interactions between LYAR and RNP subunits can be mediated by RNAs. To test our hypothesis, the same experiments were conducted using RNase A-treated cell lysates. The host protein PLSCR1, which is reported to interact with NP of A/WSN/33 (WSN, H1N1) in an RNA-independent manner (47), was used as a control. Results showed that PLSCR1 was coprecipitated with PR8 NP with or without RNase A treatment (Fig. 2A and B). In contrast, all of the RNP subunits failed to coprecipitate LYAR under RNase A treatment (Fig. 2B), indicating that LYAR interacts with RNP components in an RNA-dependent manner. The interaction between RNP components and endogenous LYAR was further studied by using influenza virus-infected A549 cells and coimmunoprecipitation with an anti-LYAR mouse antibody. The results revealed that PA, PB1, PB2, and NP were all coprecipitated by LYAR (Fig. 2C), demonstrating a real interaction between LYAR and RNP components during virus infection. Moreover, we found that RNase A treatment also disrupted the interaction between LYAR and RNP components in virus-infected cells (Fig. 2C), indicating that LYAR interaction with RNP components during virus infection is mediated by RNAs. To investigate the interaction between LYAR and the vRNP complex, we used a vRNP reconstitution system to construct vRNPs in which the NP was HA tagged. Previous studies claim that because NP and PA do not interact directly, their coprecipitation can only occur in the context of a vRNP (14, 48), which is also confirmed by our studies, which showed that NP did not coprecipitate PA when other vRNP subunits, including PB1, PB2, and vRNA, were absent (Fig. S6A and B). Our results showed that PA was specifically coprecipitated by HA-tagged NP, indicating that the vRNP complexes were immunoprecipitated, and LYAR was also detected in these immunoprecipitated complexes (Fig. 2D), indicating that LYAR associates with the reconstituted vRNPs. Additionally, when the lysine-rich region of LYAR (CTD, for C-terminal domain), which has RNA binding ability, was deleted, the interaction between LYAR (NTD, for N-terminal domain) and NP in the context of the vRNP was disrupted (Fig. 2D). Taken together, the results demonstrate that LYAR interacts with each RNP component and the C-terminal domain of LYAR plays a critical role in interaction with vRNP.

**FIG 3:**
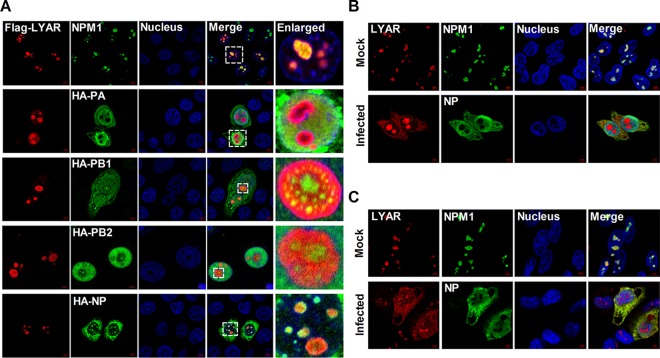
Colocalization of LYAR and RNP components. (A) Colocalization of LYAR and RNP components in transfected cells. HeLa cells cultured on slides were cotransfected with Flag-LYAR and HA-PA, -PB1, -PB2, -NP, or vector (HA). Cells were fixed at 24 h posttransfection and stained for LYAR (red) and viral proteins (green) or endogenous NPM1 (green) using the anti-Flag mouse antibodies and anti-HA rabbit antibodies or anti-NPM1 rabbit antibodies, followed by immunostaining with the Alexa Fluor 594-conjugated AffiniPure goat anti-mouse secondary antibodies and Alexa Fluor 488-conjugated AffiniPure goat anti-rabbit antibodies. DAPI was used to stain for the nucleus (blue). The boxed region was enlarged and is shown on the right. (B and C) Colocalization of LYAR and RNP components in IAV-infected cells. HeLa cells (B) and A549 cells (C) were left uninfected (mock) or infected with the PR8 H1N1 virus (MOI of 2) for 6 h, and confocal microscopy was performed using an anti-LYAR mouse antibody (red) and anti-NP rabbit antibody (green) or anti-NPM1 rabbit antibody (green), followed by immunostaining with the Alexa Fluor 594-conjugated AffiniPure goat anti-mouse secondary antibodies and Alexa Fluor 488-conjugated AffiniPure goat anti-rabbit antibodies. The nuclei were visualized by DAPI (blue). For all of these experiments, fluorescence was examined with a confocal microscope (LSM 880; Zeiss). Images are representative of three independent experiments. Scale bar, 5 μm.

The colocalizations between LYAR and RNP components were further examined using immunofluorescence and confocal microscopy. In HeLa cells cotransfected with Flag-LYAR and either HA-PA, -PB1, -PB2, or -NP, LYAR localized to the nucleoli and colocalized with NPM1 (nucleolar maker) when expressed alone, and it colocalized to the nucleoli when expressed with PB1, PB2, or NP but diffused in the nucleoplasm when expressed with PA (Fig. 3A). In uninfected HeLa (Fig. 3B) and A549 cells (Fig. 3C), endogenous LYAR appeared as foci and localized in inside the region of NPM1 in the nucleoli. Interestingly, in cells infected with IAV PR8 for 6 h, some LYAR proteins were detected in nucleoplasm and cytoplasm and colocalized with NP (Fig. 3B and C), indicating that IAV infection triggers translocation of LYAR. Taken together, these data suggest that LYAR proteins have a role in IAV transcription and replication or vRNP nuclear export.

### IAV infection increases the expression of LYAR.

**FIG 4:**
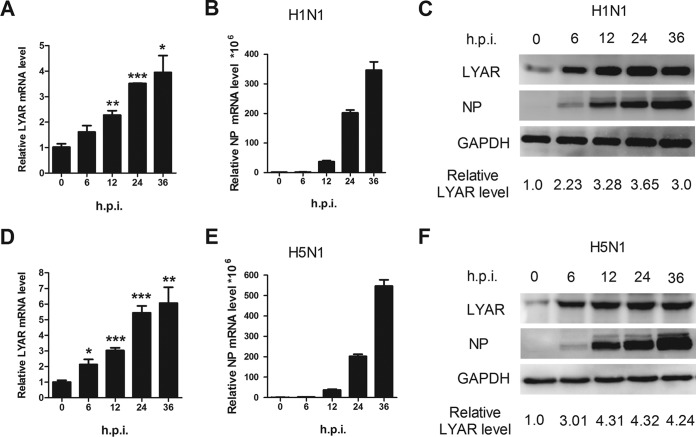
Expression level of LYAR during IAV infection. A549 cells were left uninfected or were infected with the PR8 H1N1 virus (A to C) or HM H5N1 virus (D to F) at an MOI of 0.01. Samples were collected at 0, 6, 12, 24, and 36 hpi, followed by qRT-PCR and Western blotting to determine the mRNA (A and B and D and E) and protein levels (C and F) of NP and LYAR, respectively. The mRNA level was normalized to the 18S rRNA level. The data are presented as means ± SD from three independent experiments (*, *P* < 0.05; **, *P* < 0.01; ***, *P* < 0.001; all by two-tailed Student's *t* test). For Western blot analysis, GAPDH was used as a loading control. The band intensities were quantified by ImageJ (NIH), and the relative LYAR levels (LYAR/GAPDH) are shown below.

To further explore the role of LYAR in the IAV life cycle, we next investigated the progress of expression of LYAR during the course of virus infection. A549 cells were infected with either the PR8 H1N1 or the HM H5N1 virus, and then the mRNA and protein levels of LYAR as well as the viral NP protein were analyzed. The data showed that the mRNA level of LYAR increased in both PR8 H1N1 (Fig. 4A) and HM H5N1 (Fig. 4D) virus-infected cells, which is correlated with virus replication during the infection course (Fig. 4B and E), suggesting that IAV infection upregulates the expression of LYAR at the transcriptional level. We noted that the protein levels of LYAR gradually increased until 24 h postinfection of both viruses (Fig. 4C and F) and were not consistent with the changes in mRNA levels. This may be due to protein degradation caused by cell apoptosis at late time points of virus infection. These results indicate that the expression of LYAR is increased during IAV infection, especially during the early period, implying a role of LYAR in IAV replication.

### Knockdown of LYAR reduces IAV replication.

**FIG 5:**
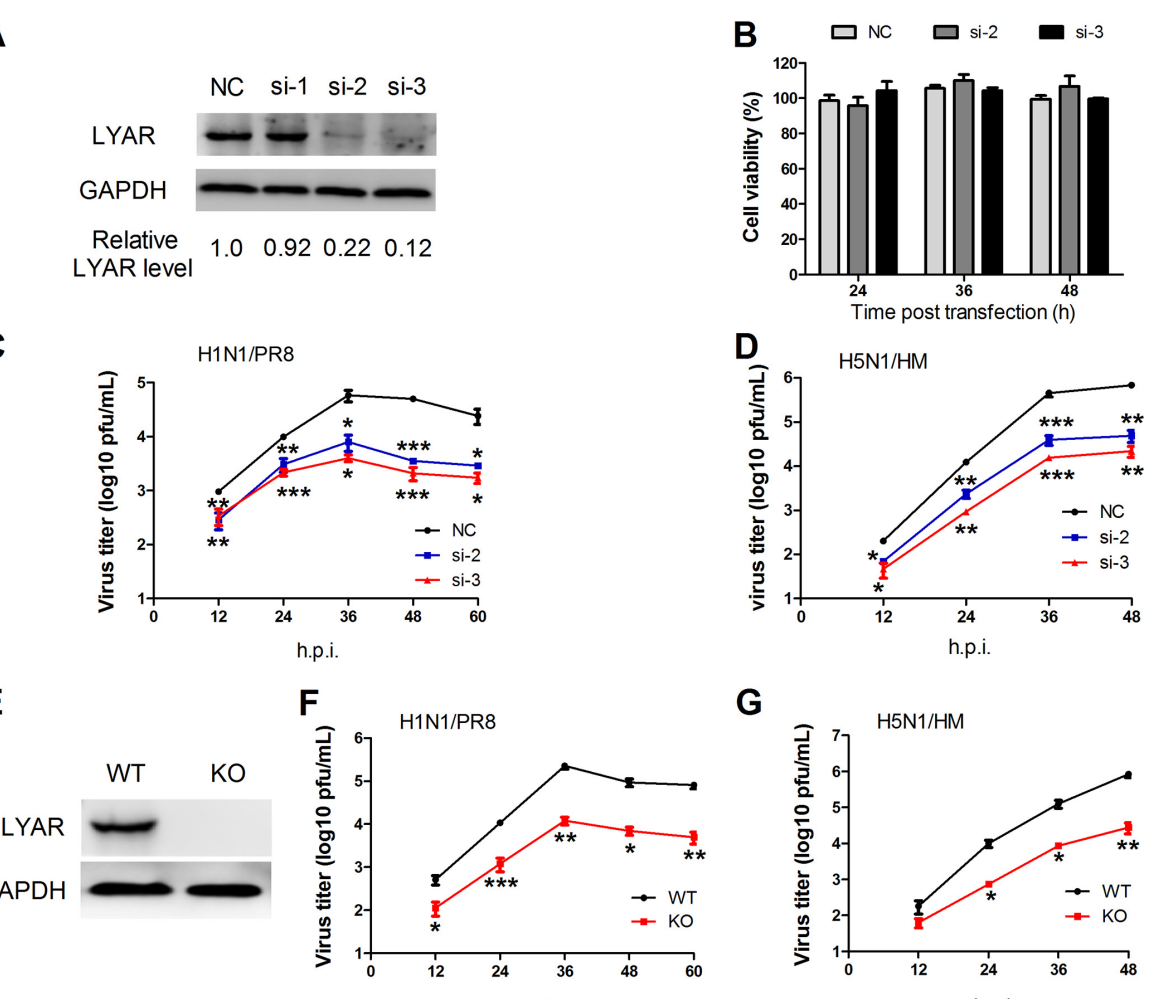
Effect of LYAR knockdown on IAV replication. (A) The silencing efficiency of LYAR-specific siRNAs. A549 cells were transfected with three individual siRNAs targeted to LYAR (si-1, si-2, and si-3) or nontarget siRNA (NC) for 36 h, followed by Western blotting to detect the protein level of LYAR. GAPDH served as a loading control. The band intensities were quantified with ImageJ, and the relative LYAR levels are shown below. (B) The effect of si-LYAR on A549 cell viability. A549 cells were treated with LYAR siRNA (si-2 and si-3) or negative-control siRNA. Cell viability was measured by CCK-8 assay at the indicated time points posttransfection. (C and D) Growth curves of IAV in LYAR-silenced and mock-treated cells. A549 cells were transfected with siRNA targeted to LYAR (si-2 and si-3) or nontarget siRNA (NC) for 24 h and then infected with the PR8 H1N1 virus (C) or HM H5N1 (D) virus at an MOI of 0.01. Cell supernatants were collected at the indicated time points postinfection. Virus titers were determined by plaque assay on MDCK cells (means ± SD from three independent experiments) (*, *P* < 0.05; **, *P* < 0.01; ***, *P* < 0.001; all by two-tailed Student's *t* test). (E) Generation of LYAR-KO A549 cells. LYAR-KO A549 cells were generated by using the CRISPR/Cas9 system. LYAR knockout was confirmed by Western blotting with an anti-LYAR mouse antibody. (F and G) Virus replication in LYAR-KO A549 cells. LYAR-KO A549 cells (KO) or wild-type A549 cells (WT) were infected with either PR8 H1N1 virus (F) or HM H5N1 virus (G) at an MOI of 0.01. Virus titers were determined by plaque assay on MDCK cells (means ± SD from three independent experiments) (*, *P* < 0.05; **, *P* < 0.01; ***, *P* < 0.001; all by two-tailed Student's *t* test).

vRNPs are responsible for viral genome transcription and replication, and the LYAR interaction with each vRNP subunit as well as it expression level increasing with IAV replication suggest that LYAR plays a critical role in IAV replication. To determine its possible role in IAV replication, three specific siRNAs (si-1, si-2, and si-3) targeting LYAR were used to knock down LYAR in A549 cells. The silencing efficiency of LYAR-targeting siRNAs detected by Western blotting showed that LYAR protein levels in si-2- and si-3-treated A549 cells were significantly decreased compared to those of the negative control (Fig. 5A); therefore, these two siRNAs were used in our further experiments. Since LYAR can regulate cell growth, the siRNA-mediated knockdown of LYAR may influence cell viability, resulting in effects on virus replication. We determined the effect of LYAR silencing on cell viability in A549 cells and showed that the cell viability of si-LYAR-treated cells and mock-treated cells were comparable at 24, 36, and 48 h posttransfection (Fig. 5B), indicating that LYAR silencing does not have effects on A549 cell viability. Growth kinetics of both PR8 H1N1 and HM H5N1 viruses showed that virus titers of both viruses were significantly reduced in LYAR-silenced cells, in contrast to that in control cells (Fig. 5C and D), indicating that knockdown of LYAR reduces IAV replication. In addition, the LYAR-KO A549 cells were produced and confirmed by Western blotting (Fig. 5E) to determine the effect of LYAR on IAV replication. Results showed that virus titers of both PR8 H1N1 and HM H5N1 viruses were dramatically decreased in the LYAR-KO A549 cells compared with those in the WT A549 cells (Fig. 5F and G), indicating that LYAR does play a critical role in IAV replication.

To examine whether LYAR specifically regulates IAV replication, we explored the effect of LYAR on two RNA viruses with a cytoplasmic replication cycle (vesicular stomatitis virus [VSV] and Japanese encephalitis virus [JEV]). Results showed that siRNA treatment significantly suppressed the replication of the recombinant VSV-GFP virus in A549 cells, which was determined by detecting GFP expression by Western blotting and was visualized by fluorescence microscopy (Fig. S7A). The effect of LYAR knockdown on JEV replication was determined by measuring the mRNA level of core protein C. The results showed that the mRNA level of JEV core protein C was significantly reduced in LYAR-silenced cells compared to that in control cells (Fig. S7B), indicating that LYAR knockdown inhibits JEV replication. These results indicate that LYAR regulates the replication of both nuclear and cytoplasmic replicating RNA viruses.

### LYAR facilitates viral RNA synthesis.

**FIG 6:**
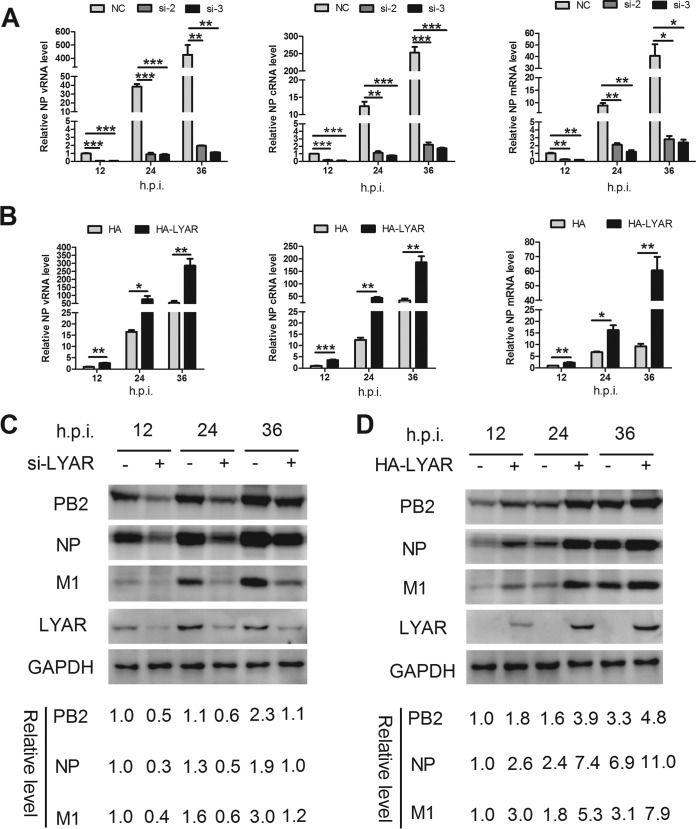
Effect of LYAR on IAV RNA synthesis. (A) The effect of LYAR silencing on IAV RNA synthesis during infection. A549 cells were transfected with LYAR siRNA (si-2 and si-3) or negative-control siRNA (NC) and then infected with the PR8 H1N1 virus (MOI of 0.01). Samples were collected at 12, 24, and 36 hpi. The levels of NP RNAs (vRNA, cRNA, and mRNA) were determined by qRT-PCR. The viral RNA levels were normalized to the 18S rRNA level (means ± SD from three independent experiments) (*, *P* < 0.05; **, *P* < 0.01; ***, *P* < 0.001; all by two-tailed Student's *t* test). (B) The effect of LYAR overexpression on NP RNA synthesis during infection. A549 cells were transfected with HA-LYAR or HA, and other procedures were the same as those described for panel A (means ± SD from three independent experiments) (*, *P* < 0.05; **, *P* < 0.01; ***, *P* < 0.001; all by two-tailed Student's *t* test). (C and D) The effect of LYAR silencing (C) and overexpression (D) on IAV protein synthesis. A549 cells were treated and infected as described above, and Western blotting was done to determine the protein levels of PB2, NP, M1, and LYAR. GAPDH was used as a loading control. The band intensities were quantified with ImageJ, and the relative PB2, NP, and M1 levels (PB2, NP, or M1/GAPDH) are shown below.

To determine the mechanisms of LYAR-influenced IAV replication, we investigated the effects of LYAR on viral RNA and protein syntheses. LYAR-silenced A549 cells were infected with the PR8 virus, and the vRNA, cRNA, and mRNA levels of NP and the protein levels of PB2, NP, and M1 were determined. The levels of all three species of viral RNA were significantly lowered in LYAR-silenced cells compared to those of the control cells at 12, 24, and 36 hpi (Fig. 6A). As expected, expression levels of PB2, NP, and M1 proteins were reduced in LYAR-silenced cells, in contrast to those in the control cells at all tested time points (Fig. 6C). In contrast, the levels of all three species of viral RNA, as well as PB2, NP, and M1 proteins, were significantly increased in cells overexpressing LYAR (Fig. 6B and D). These data indicate that LYAR promotes viral RNA and protein synthesis.

**FIG 7:**
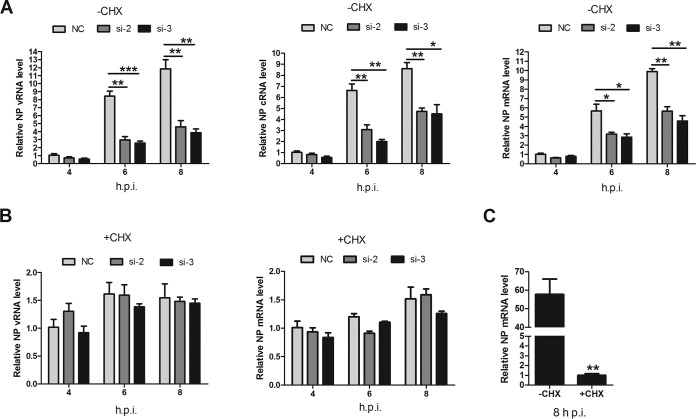
Effect of LYAR on IAV primary transcription and genome replication. (A and B) Effect of LYAR silencing on NP RNA synthesis in cells treated with CHX or left untreated. A549 cells were mock treated with DMSO (A) or treated with 100 μg/ml CHX (B) for 1 h and then infected with the PR8 H1N1 virus (MOI of 1.0). NP vRNA, cRNA, and mRNA levels in mock-treated cells and vRNA and mRNA levels in CHX-treated cells were measured at 4, 6, and 8 hpi. NP RNAs levels were normalized to the 18S rRNA level (means ± SD from three independent experiments) (*, *P* < 0.05; **, *P* < 0.01; ***, *P* < 0.001; all by two-tailed Student's *t* test). (C) A comparison of NP mRNA levels in cells infected with virus for 8 h in the presence or absence of CHX (means ± SD from three independent experiments) (**, *P* < 0.01 by two-tailed Student's *t* test).

IAV genome primary transcription is independent of *de novo* viral protein synthesis, while viral genome replication requires newly synthesized viral proteins (34). Therefore, we investigated the effects of LYAR on viral RNA genome transcription and replication separately. CHX, an inhibitor of protein synthesis in eukaryotic cells, was used to block the synthesis of viral proteins, thereby inhibiting the synthesis of cRNA and the replication of vRNA but not the level of primary transcription from incoming vRNPs (49). In LYAR-silenced cells without CHX, vRNA, cRNA, and mRNA levels were significantly reduced at 6 and 8 hpi compared to those in mock-silenced cells (Fig. 7A), whereas in the presence of CHX, similar levels of vRNA and mRNA were detected in both virus-infected LYAR-silenced cells and mock-silenced cells (Fig. 7B), suggesting that LYAR does not participate in primary transcription. To verify the inhibition of CHX treatment on virus multiplication, NP mRNA levels in CHX-treated and untreated infected cells were determined, and the results showed that NP mRNA levels were reduced approximately 60-fold after CHX treatment (Fig. 7C). Taken together, these findings demonstrate that LYAR facilitates RNA synthesis after primary transcription.

### LYAR enhances viral polymerase activity.

**FIG 8:**
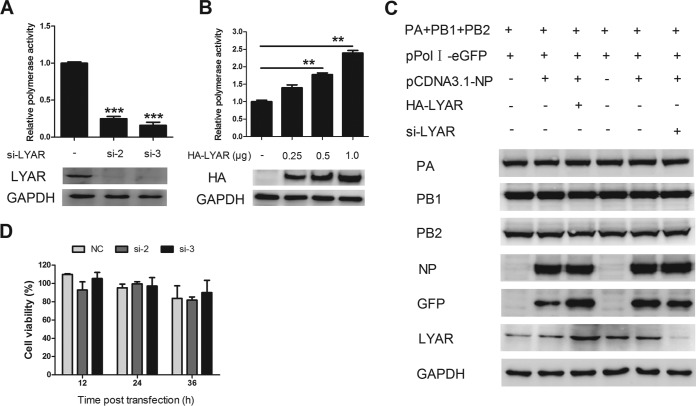
Effect of LYAR on IAV polymerase activity. (A) Effect of LYAR silencing on viral polymerase activity. HEK293T cells were cotransfected with vRNP reconstitution plasmids and Renilla together with si-LYAR (si-2 and si-3) or si-NC. Luciferase activity was measured at 24 h posttransfection, and Renilla luciferase was used as an internal control (means ± SD from three independent experiments) (***, *P* < 0.001 by two-tailed Student's *t* test). (B) Effect of LYAR overexpression on viral polymerase activity. Cells were treated as described for panel A, except cells were also transfected with HA-LYAR (0.25, 0.5, and 1.0 μg) (means ± SD from three independent experiments) (**, *P* < 0.01 by two-tailed Student's *t* test). (C) Effects of LYAR silencing and overexpression on the expression of RNP components. HEK293T cells were transfected with the indicated plasmids as described above, except cells were transfected with pPolI-eGFP instead of pPolI-luc. Protein expression of individual RNP components LYAR and GFP was analyzed by Western blotting. GAPDH was used as a loading control. (D) The effect of LYAR silencing on HEK293T cell viability. HEK293T cells were treated with si-LYAR (si-2 and si-3) or si-NC, and cell viability was measured by using CCK-8 assay at the indicated time points posttransfection.

Since LYAR facilitates viral RNA synthesis in IAV-infected cells, we further determined whether LYAR regulates viral RNA synthesis by influencing polymerase activity. For this purpose, a well-established minireplicon assay was applied to examine the effect of LYAR on the polymerase activity of PR8 H1N1 virus. HEK293T cells were cotransfected with si-LYAR (si-2 and si-3) or HA-LYAR (increasing amount of LYAR), together with plasmids encoding PB1, PB2, PA, and NP, as well as a PolI-driven RNA expression plasmid encoding the NS vRNA segment. The data showed that knockdown of LYAR resulted in an approximately 80% reduction of the polymerase activity (Fig. 8A), while overexpressing LYAR led to increased polymerase activity in a dose-dependent manner (Fig. 8B). To determine whether the LYAR-promoted polymerase activity is due to an increase of the expression levels of the RNP subunits, the expression levels of the RNP components were determined. Western blot data showed that the expression levels of of all the RNP subunits, including PA, PB1, PB2, and NP, were unchanged (Fig. 8C), although the GFP expression driven by PolI was increased in LYAR-overexpressed cells and decreased in LYAR-silenced cells. These results indicate that the polymerase activity enhanced by LYAR is not caused by increasing expression of RNP subunits. To assess the viability of HEK293T cells treated with si-LYAR, a cell-counting kit 8 (CCK-8) assay was used to measure cell viability. Results showed that there was no significant difference between LYAR knockdown cells and control cells at any tested time point (Fig. 8D). These data indicate that LYAR promotes viral polymerase activity, thereby facilitating viral RNA synthesis.

### LYAR facilitates viral RNP assembly.

**FIG 9:**
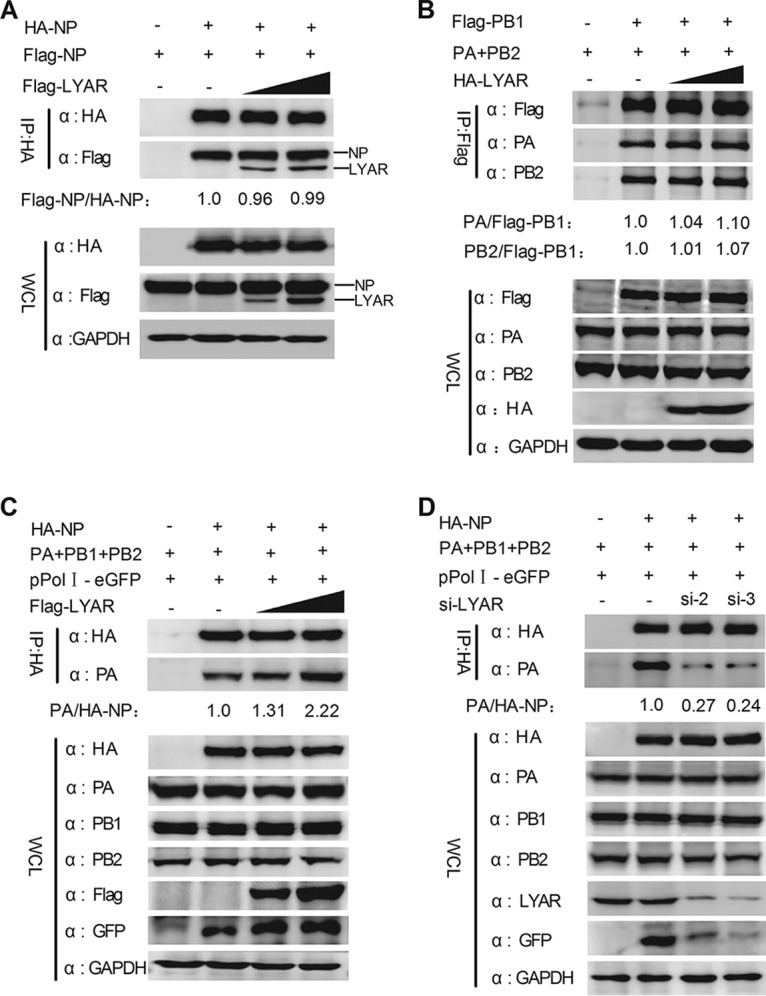
Effect of LYAR on viral RNP assembly. (A) The effect of LYAR on NP oligomerization. HEK293T cells were transfected with Flag-LYAR (0, 0.5, and 1.0 μg), Flag-NP, and HA-NP or HA. Cells were then lysed at 24 h posttransfection, and Co-IP was performed using an anti-HA antibody followed by Western blotting. The band intensities were quantified, and relative precipitated Flag-NP/HA-NP ratios are shown below. (B) Effect of LYAR on 3P formation. HEK293T cells were transfected with pCDNA3.1-PA, pCDNA3.1-PB2, HA-LYAR (0, 0.5, 1.0 μg), and Flag-PB1 or Flag. Cells were then treated as described above, and Co-IP was performed using an anti-Flag antibody. The immune complexes were analyzed by Western blotting using antibodies against PA, PB2, and Flag, respectively. The band intensities were quantified, and relative precipitated PA/Flag-PB1 and PB2/Flag-PB1 ratios are shown below. (C and D) The effect of LYAR on vRNP assembly. HEK293T cells were transfected with the vRNP reconstitution plasmids together with Flag-LYAR (0, 0.5, and 1.0 μg) (C) or si-LYAR (D), and then Co-IP was performed using an anti-HA antibody followed by Western blotting. The relative precipitated PA/HA-NP ratios are shown below. For all experiments, the band intensities were analyzed using the software ImageJ (NIH). GAPDH was used as a loading control.

Influenza virus genome replication depends on the assembly of progeny vRNPs by newly translated NP and polymerase complex proteins. We therefore determined whether LYAR participates in the viral RNP assembly process, specifically, NP oligomerization (NP self-association), 3P (PB1, PB2, and PA) formation, and viral RNP (viral RNAs, NP, and 3P) formation. The effect of LYAR on NP self-association was tested by coimmunoprecipitation of HA-NP with Flag-NP. The data showed that HA-NP associated with Flag-NP equally with or without LYAR overexpression (Fig. 9A), indicating that LYAR does not affect NP oligomerization. The effect of LYAR on 3P formation was also determined by coimmunoprecipitation, and Flag-PB1 was used as bait to immunoprecipitate PA and PB2. We showed that PA and PB2 were precipitated by PB1 equally with or without HA-LYAR (Fig. 9B), demonstrating that LYAR does not affect 3P formation, as NP directly interacts with PB1 and PB2 but not PA, and the NP-PA interaction occurs only in the context of a vRNP (14, 48). Therefore, a vRNP reconstitution system in which the NP was HA tagged was used to form the functional viral RNPs, and the Co-IP experiment was performed by using an anti-HA antibody. The amount of PA coprecipitated by HA-NP represents the efficiency of vRNP assembly. The data showed the amount of PA precipitated by HA-NP was significantly increased with an increasing amount of Flag-LYAR (Fig. 9C). Moreover, HA-NP coprecipitated much less PA in LYAR-silenced cells than the negative-control cells (Fig. 9D). The expression of GFP driven by vRNP was enhanced in LYAR-overexpressed cells and reduced in LYAR-silenced cells compared to that in the control cells, while the expression of all RNP components was almost not changed (Fig. 9C and D), suggesting that LYAR directly affects vRNP assembly. Taken together, these results provide evidence that LYAR facilitates vRNP assembly.

### LYAR knockdown leads to nuclear retention of vRNPs.

**FIG 10:**
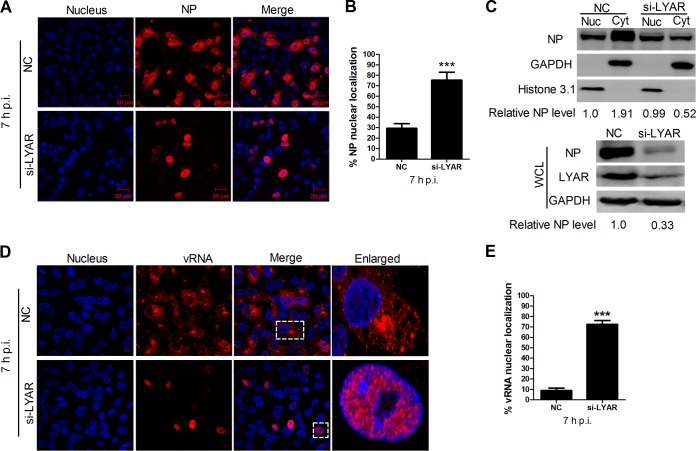
Effect of LYAR on vRNP nuclear export. (A and B) Confocal microscopy analysis of the nucleocytoplasmic distribution of NP in virus-infected LYAR knockdown cells. A549 cells transfected with si-LYAR (si-3) or si-NC were infected with the PR8 H1N1 virus (MOI of 2.0). At 7 hpi, NP was detected by IFA using an anti-NP antibody (red), and images were acquired by confocal microscopy (LSM 510; Zeiss). Scale bar, 20 μM. Images are representative of three independent experiments. Six images in a random field of view from each sample were scored by the Cell Counter plugin of ImageJ (NIH). (B) The ratios of NP nuclear-retained cells to total infected cells were analyzed from three independent experiments (means ± SD from three independent experiments) (***, *P* < 0.001 by two-tailed Student's *t* test). (C) Western blot analysis of the distribution of NP in the cytoplasmic and nuclear fractions in virus-infected LYAR knockdown cells. A549 cells were treated as described for panel A. Cells were harvested and subjected to nuclear and cytoplasmic fractionation. Western blotting using an anti-NP antibody to determine the NP content of the nuclear (Nuc) and cytoplasmic (Cyt) fractions (upper panel) and whole-cell lysates (WCL) (lower panel). The silencing efficiency of LYAR was also detected (right). Histone 3.1 was used as a nuclear loading control and marker and GAPDH as a cytosolic loading control and marker. The band intensities were analyzed by ImageJ (NIH), and the relative NP levels (NP/GAPDH or Histone 3.1) are shown below. (D and E) Confocal microscopy analysis of the nucleocytoplasmic distribution of vRNAs in virus-infected LYAR knockdown cells. A549 cells were transfected with si-LYAR (si-3) or si-NC and then were infected with the PR8 H1N1 virus (MOI of 2.0). At 7 hpi, cells were subjected to *in situ* hybridization assays using Quasar 570 (red)-labeled vRNA-specific probes targeting the M gene. The boxed region was enlarged and is shown on the right. Scale bar, 10 μm. Images are representative of three independent experiments. Six images in a random field of view from each sample were scored by the Cell Counter plugin of ImageJ (NIH). (E) The ratios of vRNA nuclear-retained cells to total infected cells were analyzed from three independent experiments (means ± SD from three independent experiments) (***, *P* < 0.001 by two-tailed Student's *t* test).

Influenza vRNPs are assembled in the nucleus and subsequently exported to the cytoplasm as a complex, where new viral particles are assembled. Because LYAR silencing has been shown to significantly inhibit IAV replication, whether LYAR knockdown has effects on the nuclear export of vRNPs was determined in A549 cells infected with the PR8 H1N1 virus. Results showed that approximately 30% of mock-silenced infected cells (NC) displayed NP nuclear localization at 7 hpi (Fig. 10A and B), indicating that the nuclear export of vRNPs had occurred in most of the infected cells. In contrast, nearly 80% of LYAR-silenced infected cells (si-LYAR) had an NP nuclear localization (Fig. 10A and B), indicating vRNP nuclear export was largely delayed due to LYAR silencing. Furthermore, the results of Western blotting on nuclear and cytoplasmic fractions of these infected cells were consistent with the indirect immunofluorescence assay (IFA) data, i.e., the NP was predominantly in the cytoplasmic fraction of the mock-silenced cells, in contrast to that in the nuclear fraction of the LYAR-silenced cells (Fig. 10C). Meanwhile, a significant reduction of NP protein level was detected in the whole-cell lysis of LYAR-silenced cells (Fig. 10C), indicating the viral protein synthesis was also inhibited when LYAR was knocked down. In addition, the distribution of vRNAs was detected by using an *in situ* hybridization assay. The results showed that vRNAs of the viral M segment were markedly retained in the nucleus in LYAR-silenced infected cells at 7 hpi (Fig. 10D and E), and the total amounts of M vRNAs in the LYAR-silenced cells were markedly reduced compared to those in the control cells (Fig. 10D), indicating LYAR knockdown inhibits the nuclear export of vRNPs as well as vRNA synthesis. Collectively, these results demonstrate that LYAR knockdown results in a marked retention of vRNPs in the nucleus.

## DISCUSSION

Transcription and replication of IAV is performed by vRNPs in the nucleus and is reliant on host proteins. In past decades, proteomics-based screens have been carried out to identify cellular factors interacting with viral NP, polymerase, and RNP, revealing hundreds of candidate proteins that could regulate viral RNA synthesis (28, 29, 31, 35, 50). However, the exact roles of these host factors involved in the IAV life cycle remain largely undetermined. Here, we identified 80 cellular proteins that copurified with the reconstituted vRNPs using AP-MS. Of these identified proteins, some have been shown to interact with the full vRNP complex, polymerase complex, or RNP components and play roles in IAV replication. For example, DDB1, NCL, EEF1A1, TUBB, and RPL19 were identified by Mayer et al. as vRNP binding partners (28). DDB1 has been shown to interact with reconstituted vRNP (28) and is required for efficient activity of both H1N1 and H5N1 virus polymerase (31). NCL interacts with IAV nucleoprotein and contributes to vRNP nuclear trafficking and efficient viral replication (26). In addition, other proteins, such as FKBP4, KHSRP, HNRNPC, MIF, and TUFM (51–56), have been shown to be involved in the influenza virus life cycle. In particular, TUFM interacts with PB2 and acts as a host restriction factor, impeding avian-like IAV (with avian signature) replication in human cells in a manner that correlates with autophagy (57). These studies indicate that vRNP-interacting host proteins regulate influenza virus replication in different manners, and understanding interplay between host factors and vRNP has the potential to reveal underlying mechanisms of virus-host interactions that can be used to develop novel antivirals.

IAV utilizes host nuclear machineries to complete various important life stages, such as transcription and replication, mRNA splicing and trafficking, and vRNP nuclear export. Previous studies have shown that there is an intense functional interplay between IAV and nucleoli (25, 58). IAV vRNP hijacks the nucleolar proteins, such as NCL, NPM1, RRP1B, and some ribosomal proteins, to facilitate virus transcription and replication, or vRNP nuclear export (25–28), of which rRNA processing 1 homolog B (RRP1B) interacts with PB1 and PB2, enhancing viral transcription (27). NPM1 interacts with reconstituted vRNP, promoting viral polymerase activity (28). In this study, we show that among the identified candidate proteins, LYAR displays the strongest inhibitory effect on virus replication and polymerase activity when it is silenced. Further studies show that LYAR interacts with the four components of RNP in an RNA-dependent manner and also associates with the reconstituted vRNP, indicating that LYAR associates with vRNP complex during virus infection. Moreover, LYAR partly redistributes from nucleoli to nucleoplasm and cytoplasm, and knockdown of LYAR leads to marked delay of vRNP nuclear export, suggesting that LYAR proteins associate with vRNP to form a complex in the nucleus and then are transported to the cytoplasm, which would provide an efficient means of rapidly establishing vRNP nuclear export after assembly. Additionally, LYAR can directly interact with NCL (60) and colocalize with NPM1 in nucleoli, providing a possibility for them to form a complex during virus infection. Therefore, NCL and NPM1 may cooperate with LYAR in these processes because they are also recruited to the nucleoplasm or cytoplasm during IAV infection and are utilized by vRNPs for virus transcription and replication or vRNP nuclear export (26, 28, 31).

Interestingly, some LYAR proteins were found to be outside the nucleoli upon virus infection, which may be caused by IAV-induced nucleolar stress. It has been reported that IAV can induce nucleolar stress (61), and LYAR diffuses to the nucleoplasm upon ActD (actinomycin D, a drug used to induce nucleolar stress) treatment (43). Similar to the ActD treatment, LYAR also diffuses into the nucleoplasm when coexpressed with PA, suggesting that the recruitment of LYAR by vRNP to the nucleoplasm is due to the nucleolar stress induced by PA. However, whether PA can induce the nucleolar stress needs to be investigated in future studies. Another possibility is that the RNP components directly recruit LYAR from nucleolus to nucleoplasm because they have been shown to localize to the nucleolus during virus infection (62). Moreover, the expression of LYAR is enhanced upon virus infection, and the alteration of expression and distribution of LYAR will definitely affect LYAR functions, thereby influencing nucleolus functions, which may in turn facilitate virus replication.

IAV primary transcription begins as soon as the vRNPs reach the nucleus, followed by a transition to genome replication and additional transcription, which requires *de novo* assembly of viral RNPs. Both viral and host factors are involved in this transition. For example, viral factors include NS1, NEP, small viral RNA (svRNA), and *trans*-activating polymerase (63–66); host factors include FMRP, which promotes vRNA replication by stimulating viral RNP assembly (67), and host protein kinase C (PKC) members, which regulate vRNP assembly by affecting NP oligomerization (14). We show that LYAR promotes viral RNA synthesis during virus infection but does not affect primary transcription, suggesting that LYAR facilitates genome replication and consequently additional transcription. Further studies demonstrate that LYAR promotes vRNP assembly, which is required for genome replication. The viral RNPs are formed by the newly synthesized polymerase, NP oligomers, and either cRNA (cRNPs) or vRNA (vRNPs) (9). Within the viral RNP complex, NP associates with itself, with RdRp, and with RNAs (9). In this study, LYAR overexpression does not affect NP oligomerization, an important factor affecting viral RNP assembly, which has been shown to be regulated by phosphorylation (14, 68). In addition, the polymerase formation required for vRNP assembly is not affected by LYAR. However, LYAR enhances the interaction between NP and PA in the context of vRNPs, revealing that LYAR facilitates either NP interactions with the polymerase or NP recruitment to nascent vRNA and cRNA during viral RNP assembly. Therefore, LYAR promotes viral genome replication by facilitating viral RNP assembly and consequently promoting virus replication. In addition, LYAR knockdown results in nuclear retention of vRNPs, which may be due to the insufficient assembly of vRNPs or its direct participation in vRNP nuclear export. Whether LYAR directly involves vRNP nuclear export should be investigated in the future. On the other hand, we cannot exclude the possibility that LYAR plays additional roles in the virus life cycle, aside from regulating vRNP functions. Based on the evidence that LYAR knockdown inhibits replication of both VSV and JEV, LYAR is involved in the regulation of multiple virus replication events. Considering that both VSV and JEV are sensitive to type I interferon, LYAR might regulate their replication by modulating innate immunity signal pathways. Moreover, it may be attributed to the nucleolar functions which LYAR is involved in, since both nuclear and cytoplasmic viruses utilize the nucleolar functions during their replication (59). Therefore, studies will be required to determine how LYAR regulates replication of these viruses.

In summary, we have identified 80 putative vRNP-interacting partners by using an AP-MS experiment and demonstrated that LYAR is a novel vRNP binding protein that is critical for IAV replication. We further reveal that LYAR enhances vRNP assembly as an underlying mechanism of LYAR-regulated IAV RNA synthesis. Our studies uncover the function of LYAR in IAV replication and provide new insights into the regulation of the nucleolar factors in IAV transcription and replication.

## MATERIALS AND METHODS

### Cells and viruses.

Human embryonic kidney 293T cells (HEK293T) and Madin-Darby canine kidney (MDCK) cells were purchased from the ATCC (American Type Culture Collection, Manassas, VA, USA) and maintained in Dulbecco's modified Eagle's medium (DMEM) (Gibco, NY, USA) supplemented with 10% fetal bovine serum (FBS) (PAN-Biotech, Germany). The Henrietta Lacks strain of cancer cells (HeLa) and adenocarcinomic human alveolar basal epithelial cells (A549) were purchased from the ATCC and maintained in RPMI 1640 and F12 media (HyClone, Beijing, China), respectively, supplemented with 10% FBS. All cells were cultured at 37°C in a 5% CO_2_ humidified atmosphere. The IAVs used in this study were A/Puerto Rico/8/1934 (PR8, H1N1) and A/duck/Hubei/Hangmei01/2006 (HM, H5N1). All viruses were amplified using 10-day-old embryonic chicken eggs and then titrated by determining log_10_ PFU/ml values on MDCK cells. All cell experiments with H5N1 virus were performed in an animal biosafety level 3 (BSL-3) laboratory. This study was carried out in accordance with the recommendations for BSL-3 of Huazhong Agricultural University (HZAU). The protocol was approved by the BSL-3 laboratory of HZAU. The recombinant vesicular stomatitis virus expressing green fluorescence protein (VSV-GFP) was a gift from the Harbin Veterinary Research Institute (Harbin, China). Japanese encephalitis virus (JEV) was kindly provided by Shengbo Cao (Huazhong Agricultural University, Wuhan, China).

### Plasmids and small interfering RNAs.

For construction of p3XFlag-LYAR (Flag-LYAR) and pCAGGS-HA-LYAR (HA-LYAR), the full-length cDNA of LYAR amplified by PCR was cloned into vectors p3XFlag (Flag) and pCAGGS-HA (HA), digested by BglII/XbaI and ECORI/XhoI, respectively. The Flag-tagged, HA-tagged, and nontagged pCDNA3.1 plasmids encoding PB1, PB2, PA, and NP were derived from the H1N1 (A/Puerto Rico/8/1934) virus. siRNAs targeting human gene LYAR, NCL, HNRNPR, MCDRH, PPP1CA, or RPL19, with a nontarget siRNA (si-NC) as a negative control, were synthesized by GenePharma (Shanghai, China) and used in this study. PCR primers and siRNA sequences are shown in Table S1 in the supplemental material.

### Antibodies and reagents.

Antibodies used for Western blotting, immunoprecipitation, and indirect immunofluorescence were anti-Flag M2 mouse monoclonal antibody (F3165; Sigma, USA); anti-LYAR mouse polyclonal antibody (H00055646-B01P; Abnova, China); anti-NPM1 rabbit polyclonal antibody (AP2834a; ABGENT, USA); anti-HA, -GFP, and -glyceraldehyde-3-phosphate dehydrogenase (GAPDH) mouse monoclonal antibodies (PMK013C, PKM009S, and PMK043F; PMK Bio, China); anti-Histone 3.1 polyclonal rabbit antibody (p30266; Abmart, USA); rabbit polyclonal antibodies against influenza A viral proteins PB1, PB2, PA, NP, and M1 (GTX125923, GTX125926, GTX118991, GTX125989, and GTX125928; GeneTex, USA); and Alexa Fluor 488-conjugated AffiniPure goat anti-rabbit and Alexa Fluor 594-conjugated affinipure goat anti-mouse secondary antibodies (SA00006-2 and SA00006-3; Proteintech, USA). The small-molecule compounds used in this study were CHX (cycloheximide; 100 μg/ml; 66819; Sigma, USA) and DAPI (4′,6′-diamidino-2-phenylindole dihydrochloride; 1:1,000) (C1002; Beyotime, China).

### Transfections.

Transfections were performed using Lipofectamine 2000 (Invitrogen) according to the manufacturer's instructions. Briefly, plasmids, siRNAs, and Lipofectamine were diluted to equal volumes with Opti-MEM and incubated for 5 min at room temperature. The diluted Lipofectamine and the diluted DNA (or RNA) were mixed and incubated for 20 min at room temperature. The mixture was added to cells and incubated for 6 h, and cells were then cultured in fresh medium supplemented with 10% FBS.

### Generation of LYAR-KO A549 cells.

LYAR-KO A549 cells were generated by using the CRISPR/Cas9 system as described previously (69, 70). The single guide RNA (sgRNA) sequence targeting the human LYAR gene (5′-TGAGCATCACAGATCCGAAG-3′) was cloned into lentiCRISPR v2 vector and applied for producing the recombined lentivirus. A549 cells were infected with the LYAR lentiCRISPR v2 lentivirus or the empty vector lentiCRISPR v2 lentivirus (negative control). After 48 to 60 h postinfection, puromycin (2.5 mg/ml) was added to select the positive clones. Finally, the monoclonal cells acquired by using the limiting dilution method were expanded and the knockout of LYAR was confirmed by Western blotting.

### Co-IP assay.

HEK293T cells were transfected with the indicated plasmids, and then cells were washed with cold phosphate-buffered saline (PBS) and lysed in radioimmunoprecipitation assay buffer (V900854; Sigma, USA) containing Complete protease inhibitor cocktail (B14001; Biotool, USA) at 24 to 48 h posttransfection. The lysates were pretreated with 20 μl of protein A/G agarose (sc-2003; Santa Cruz, USA) for 1 h at 4°C, and then protein A/G agarose was removed by centrifugation. Two to three μg of the indicated antibody was added to the pretreated lysates, followed by overnight incubation at 4°C. Protein A/G agarose was added to the lysates and incubated at 4°C for another 2 h with rotation. The agarose beads were collected by centrifugation and washed four times with lysis buffer. The beads were resuspended in 1× SDS loading buffer and proteins were resolved by SDS-PAGE, followed by transferring to nitrocellulose and Western blotting.

### IFA and confocal microscopy.

Indirect immunofluorescence and confocal microscopy were performed as described previously (71). Briefly, HeLa or A549 cells were fixed with 4% paraformaldehyde (PFA) for 10 min, treated with 0.2% (vol/vol) Triton X-100 for 10 min, and then incubated with 1% (wt/vol) bovine serum albumin (BSA) for 1 h at room temperature. Samples were then incubated with the indicated primary antibody for 2 h, followed by incubation with the appropriate Alexa Fluor-conjugated secondary antibody, and stained with DAPI to visualize DNA. Images were acquired using a confocal microscope (LSM510 or LSM880; Zeiss, Germany).

### Minireplicon assay.

Polymerase activity was measured by a minireplicon assay. In brief, HEK293T cells were cotransfected with pPolI-luc or pPolI-eGFP, a Renilla luciferase expression plasmid, and four RNP expression plasmids, pCDNA3.1-PB1, pCDNA3.1-PB2, pCDNA3.1-PA, and pCDNA3.1-NP. Luciferase activity was measured using a Dual-Luciferase assay kit (Promega) according to the manufacturer's protocol at 24 h posttransfection. Renilla luciferase activity was used as an internal control and to normalize transfection efficiency.

### Virus titration.

A549 cells in 12-well plates were transfected with the indicated plasmids (1 μg) or the indicated siRNA (40 pmol). At 24 h posttransfection, cells were infected with the indicated influenza virus at a multiplicity of infection (MOI) of 0.01. Viral supernatants were harvested at the indicated time points postinfection, and plaque assays were performed on MDCK cells to titrate virus titer as described previously (72).

### RNA isolation and qRT-PCR.

For quantitative reverse transcription-PCR (qRT-PCR), cells were lysed with TRIzol reagent (Invitrogen, USA), and total RNA was extracted according to the manufacturer's instructions. One to two micrograms of RNA was used to generate cDNA using reverse transcriptase (AMV XL; TaKaRa, Tokyo). Real-time PCR (Vii7A; ABI, USA) was performed using FastStart Universal SYBR green Master (Roche). The PCR conditions were 2 min at 50°C, 10 min at 95°C, and then 40 cycles of 15 s at 95°C and 1 min at 60°C. The levels of viral NP vRNA, cRNA, and mRNA were determined by using a strand-specific real-time RT-PCR as described previously (73). To ensure the specific amplification of vRNA, cRNA, and mRNA, primers complementary to each type of RNA added a tag of 18 to 20 nucleotides unrelated to the influenza virus to the 5′ end were used in reverse transcription, and the tagged cDNA was amplified by real-time PCR using the tag portion as the forward primer and a segment-specific reverse primer. 18S rRNA was used as a control for the normalization of cellular mRNA and intracellular viral RNA. The sequences of the primers used for qRT-PCR are shown in Table S1.

### Nuclear and cytoplasmic fractionation.

Subcellular fractions were extracted as described previously (74). A total of 10^6^ A549 cells treated accordingly were harvested and lysed with 100 μl of cytoplasmic extraction buffer [10 mM HEPES, 10 mM KCl, 2 mM Mg(Ac)_2_, 3 mM CaCl_2_, 340 mM sucrose, 1 mM dithiothreitol (DTT), 1 mM phenylmethylsulfonyl fluoride (PMSF), pH 7.9] on ice for 20 min, followed by the addition of NP-40 (Amresco, Solon, OH, USA) to a final concentration of 0.25% (vol/vol). Samples were then vortexed for 15 s and centrifuged for 10 min at 3,500 × *g* at 4°C. Supernatants (the cytoplasmic fraction) were collected and stored at −80°C. Pellets were dissolved in 80 μl nuclear extraction buffer [50 mM HEPES, 500 mM NaCl, 1.5 mM MgCl_2_, 0.1% (vol/vol) Trition-X100, 1 mM DTT, 1 mM PMSF, pH 7.9], incubated on ice for 10 min, and then centrifuged at 14,000 × *g* for 10 min at 4°C. Supernatants (the nuclear fraction) were collected and stored at −80°C.

### FISH.

Fluorescence *in situ* hybridization (FISH) probes labeled with the Quasar 570 fluorophore for detecting PR8 H1N1 M segment vRNA were designed by using an online probe designer (Stellaris Probe Designer version 4.2), and the sequences are presented in Table S1. The probes were purchased from Biosearch Technologies (Novato, CA, USA). For *in situ* hybridization analysis, A549 cells in a 24-well plate were fixed for 10 min with 4% PFA and then washed with PBS three times. Cells were then permeabilized with 0.2% Triton X-100 for 10 min and washed briefly. Cells next were incubated with 200 μl wash buffer A (SMF-WA1-60; Biosearch Technologies, USA) for 5 min. Wash buffer A was removed, and 200 μl hybridization buffer (SMF-HB1-10; Biosearch Technologies, USA) containing 2 μl FISH probes (final concentration, 12.5 nM) was added and incubated for 16 h at 37°C in the dark. The hybridization buffer was removed, and then cells were incubated with wash buffer A for 30 min at 37°C. DAPI was added to counterstain the nuclei, and then cells were incubated in wash buffer B (SMF-WB1-20; Biosearch Technologies, USA) for 5 min and washed briefly with PBS. Images were obtained with a confocal microscope (LSM 880; Zeiss, Germany).

### Cell viability assay.

To detect the effect of LYAR silencing on cell proliferation, the cell viability of HEK293T cells and A549 cells transfected with si-LYAR or si-NC were measured by CCK-8 activity according to the manufacturer's instructions (Dojindo Molecular Technologies). In brief, cells in 96-well plates were transfected with si-LYAR or si-NC, and cell viability was measured at 24, 36, and 48 h posttransfection. CCK-8 reagent was added to each well of a plate, and the absorbance at 450 nm was measured by a microplate reader after 1 h of incubation.

### Statistical analysis.

The data are presented as means ± standard deviations (SD) from three independent experiments. Statistical significance was determined using two-tailed Student's *t* test. A *P* value of less than 0.05 was considered statistically significant, and a *P* value of less than 0.01 was considered highly significant (*, *P* < 0.05; **, *P* < 0.01; ***, *P* < 0.001).

## Supplementary Material

Supplemental file 1

Supplemental file 2

Supplemental file 3

Supplemental file 4

Supplemental file 5

Supplemental file 6

Supplemental file 7
