# Supplementary material for: The Nucleolar Protein LYAR Facilitates Ribonucleoprotein Assembly of Influenza A Virus
Source: J Virol. 2018 Nov 12;92(23):e01042-18. doi: 10.1128/JVI.01042-18 (PMC6232469; doi:10.1128/JVI.01042-18)
Supplement: Supplemental file 7 [file zjv023184012s7.pdf]

<u>Supplemental information</u>

Materials and methods

Recombinant IAV vRNPs purification

Ten of 10 cm dishes of 80% confluent HEK293T cells were transfected with pPolI-eGFP and pCDNA-3.1-PA, pCDNA3.1-PB2, pCDNA-3.1-NP, and pFlag-PB1 or control vector (PA 1.0 μg; PB1, PB2 and pPolI-eGFP 3.0 μg; NP 6.0 μg per dish). Cells were harvested at 36 hours post transfection, followed by washing with cold phosphate-buffered saline (PBS) and pelleting at 350 x *g* for 10 min at 4 °C. Lysates were prepared by incubating cells on ice for 30 min in RIPA buffer (V900854, Sigma, USA). The soluble fraction was separated by centrifugation at 14,000 x *g* for 10 min at 4 °C. Cell lysates were pretreated with 20 μL protein A/G agarose (sc-2003, Santa Cruz, USA), then the agarose beads was removed by centrifugation. 20 μL monoclonal anti-Flag M2 affinity gel (A2220, Sigma, USA) was added to the pretreated lysates and incubated at 4 °C on a rotator for 12 h. Beads were washed 4 times with lysis buffer. Bound proteins were eluted using 0.1 M glycine-HCl (pH 3.5) at room temperature for 20 min followed by centrifugation at 1000 x *g* for 2 min at room temperature, then were immediately re-equilibrated to pH 7.5-7.9 using 3M Tris-base buffer. Samples were stored at -80 °C until used for MS analysis.

Mass spectrometry

Purified samples were subjected to nano-LC-MS/MS (nLC) analysis. A Q-Exactive mass spectrometer (Thermo Fisher Scientific) coupled with an Easy nLC (Thermo Fisher Scientific) were used to analyze the mass spectrometry data. MS/MS spectra were searched using MASCOT engine (Matrix Science, London, UK; version 2.2) against UniProt database (human

proteins and Influenza A virus H1N1 A/Puerto Rico/8/1934). For protein identification, the following options were used. Peptide mass tolerance = 20 ppm, MS/MS tolerance = 0.1 Da, Enzyme = Trypsin, Missed cleavage = 2, Fixed modification: Carbamidomethyl (C), Variable modification: Oxidation (M), filter by score ≥ 20. One unique peptide was the minimum required to make a positive protein identification. Two independent experiments were performed, and for each experiment two samples were analyzed; the control group- proteins purified from cells co-transfected with a control vector and RNP component expression plasmids, except PB1 and the RNP group- proteins purified from cells co-transfected with Flag-PB1 and RNP component expression plasmids. Samples from each experiment were analyzed on two separate occasions. Any protein identified in any control group or identified in only one group of the RNP was regarded as non-specific binding and was removed.

For the construction of the host-host protein interaction network, the STRING database (http://string-db.org) (1) was used to analyze the interactions between host proteins, and Cytoscape software (2) was used to visualize the results. GO (Gene Ontology, http://www.geneontology.org/) annotation of host proteins was performed by the Blast2Go (https/:www.blast2go.com/) software (3). KEGG (Kyoto Encyclopedia of Genes and Genomes http://www.kegg.jp/) pathway annotation of host proteins was performed by KAAS (KEGG Automatic Annotation Serve) software (4).

Results

Purification of host proteins associated with vRNP from HEK293T cells

Host-vRNP interplay takes place throughout the IAV life cycle, but especially during viral

transcription and replication. To identify the host proteins that interact with influenza vRNPs, we purified reconstituted vRNPs of PR8 H1N1 from human 293T cells using the Flag-PB1, and LC-MS/MS to determine the binding partners. To ensure the Flag-tagged PB1 RNP could successfully form a functional vRNP complex in HEK293T cell, a minireplicon assay was performed. The data showed that the polymerase activity of Flag-tagged PB1 vRNP was slightly reduced compared to untagged vRNP (**Fig. S1A**), and the GFP signal driven by untagged vRNP was similar to that of the Flag-tagged PB1 vRNP (**Fig. S1B and C**), indicating that the Flag tag had little effect on vRNP formation and activity. To ensure that the whole vRNP complex could be immunoprecipitated by Flag-PB1, a Flag affinity gel was used to purify Flag-PB1 vRNP complex followed by western blotting analysis. The data showed that all the RNP components (PA, PB1, PB2 and NP) were successfully identified in the eluates of purification (**Fig. S1D**). A schematic diagram of our study used strategy procedures was depicted in **Fig. S2A**.

HEK293T cells were transfected with reconstituted Flag-PB1 vRNP or Flag vRNP plasmids, and were lysed at 36 hours post transfection when vRNPs transcription and replication activity was indicated by strong intracellular GFP signals (**Fig. S2B**). Native Flag-PB1 vRNP complexes were isolated by one step purification using Flag affinity gel. The purified complexes (5% volume eluents) were subjected to SDS-PAGE followed by silver stain. The data showed that a number of cellular proteins co-purifying with Flag-PB1 that were absent in the negative control (**Fig. S2C**). The purified sample was also subjected to MS analysis.

Identification of vRNP interaction partners by mass spectrometry

Proteins co-purified with Flag-PB1 were identified by mass spectrometry. All the RNP

components were detected, and eighty cellular proteins were specifically identified (**Fig. S3 and Table. S2**). Of these, nineteen have been reported to interact with vRNPs, eight have been shown to interact with RdRp and forty-six have been shown to associate with one or more RNP subunits (**Table. S3**). In addition, eleven proteins interacted with both the full vRNP complex and all the four of the RNP subunits (**Table. S3**). Many identified proteins are pro-viral factors (**Table. S4**), suggesting that cellular proteins associated with vRNPs are probably required for efficient influenza virus replication.

To further understand the cellular functions of the identified host proteins, KEGG and GO analysis were performed. KEGG pathway analysis showed that these proteins mainly participate in ribosome, RNA transport, spliceosome, proteasome and phagosome pathway (**Fig. S4 and Table. S5**). GO analysis showed that the identified proteins participate in various biological processes, such as response to stimuli, transport, transcription, translation, and RNA splicing, and that they are enriched in numerous cellular components with varied molecular functions (**Fig. S5 and Table. S5**), which suggests a relationship between the host proteins and influenza virus replication. In addition, most of the bound proteins could interact with at least one of the other bound proteins, suggesting that complexes of interacting proteins may have been purified (**Fig. S4 and Table. S6**). Consistent with this, many ribosomal proteins were identified (**Fig. S4 and Table. S6**).

References

1. **Jensen LJ, Kuhn M, Stark M, Chaffron S, Creevey C, Muller J, Doerks T, Julien P, Roth A, Simonovic M, Bork P, von Mering C.** 2009. STRING 8--a global view on proteins and their functional interactions in 630 organisms. Nucleic Acids Res **37:**D412-416.
2. **Lopes CT, Franz M, Kazi F, Donaldson SL, Morris Q, Bader GD.** 2010. Cytoscape Web: an interactive web-based network browser. Bioinformatics **26:**2347-2348.
3. **Gotz S, Garcia-Gomez JM, Terol J, Williams TD, Nagaraj SH, Nueda MJ, Robles M, Talon M, Dopazo J, Conesa A.** 2008. High-throughput functional annotation and data mining with the Blast2GO suite. Nucleic Acids Res **36:**3420-3435.
4. **Kanehisa M, Goto S, Sato Y, Furumichi M, Tanabe M.** 2012. KEGG for integration and interpretation of large-scale molecular data sets. Nucleic Acids Res **40:**D109-114.

Figure legends

Figure. S1 Characterization of Flag-tagged PB1 reconstituted vRNPs. (A) Polymerase activity of untagged PB1 and Flag-PB1 vRNPs in HEK293T cells. Cells were transfected with the indicated vRNP reconstitution plasmids. Luciferase activity was measured at 24 h post transfection. The group transfected p3X-Flag or pCDNA3.1 empty vectors were negative controls. **(B-C)** Expression of GFP driven by untagged PB1 or Flag-PB1 vRNPs. HEK293T cells were transfected with pPolI-eGFP and plasmids (pCDNA3.1-PA, PB2, NP and p3X-Flag-PB1) for the expression of RNP subunits. Green fluorescence was observed at 24 h post transfection, and (C) the expression of GFP and RNP components were detected by western blotting. GAPDH served as loading control. **(D)** HEK293T cells were treated as described in (B), and Co-IP was performed using an anti-Flag antibody to detect the interactions between PB1 and the other RNP subunits. GAPDH served as loading control.

Figure. S2 Purification of the reconstituted vRNPs from HEK293T cells. (A) Schematic diagram of the AP-MS procedure. **(B)** Detection of the expression of GFP driven by reconstituted vRNPs. HEK293T cells were transfected with pPol I-eGFP together with pCDNA3.1-PA, PB2, NP and p3X-Flag-PB1 (vRNP) or p3X-Flag (Control). GFP signal was determined at 36 h post transfection. **(C)** Purified protein analyzed by SDS-PAGE and silver stain.

Figure. S3 Map of vRNP-host protein interactome. Yellow node: influenza viral protein (vRNP, PA, PB1, PB2 or NP); Green node: host protein that co-purified with vRNP. Gray edge:

interaction identified previously; Red edge: novel interaction identified in this study.

Figure. S4 Host-host proteins interactions. Yellow node: biological process performed by KEGG analysis; Blue node: host protein; Pink node: host protein localized to nucleolus. Solid line edge: host protein interactions; Dotted line: the proteins involved in the following biological process: ribosome, spliceosome, proteasome and phagosome pathway, proteins in the ER, the PI3K-AKT signaling pathway, or the cGMP-PKG signaling pathway.

Figure. S5 GO analysis of the host proteins. (A) The biological process analysis of the identified host proteins. **(B)** The molecular function of the identified host proteins. **(C)** The cellular component terms enriched in the identified host proteins.

Figure. S6 Interaction between PA and NP. (**A**) HEK293T cells were transfected with pCDNA3.1-PA and HA-NP or pCAGGS-HA. Co-IP was performed using an anti-HA antibody followed by western blotting to detect PA and NP. (**B**) HEK293T cells were transfected with pCDNA3.1-PB1, PB2, PA, and pPolI-eGFP or pCDNA3.1 together with HA-NP or HA. Cells were then treated as above and western blotting was performed to detect PA, PB1, PB2 and NP. GAPDH was used as loading control.

Figure. S7 Effect of LYAR on cytoplasmic RNA virus replication. (A) A549 cells transfected with si-LYAR or negative control for 24 h were infected with VSV-GFP (MOI=0.1). The VSV-GFP was visualized by fluorescence microscopy at 24 hpi. Original magnification

x10. The expressions of GFP and LYAR were detected by western blotting using an anti-GFP mouse antibody and anti-LYAR mouse antibody, respectively (right panel). GAPDH was used as loading control and the band intensities were quantified with the Image J. **(B)** A549 cells transfected with si-LYAR or negative control were infected with JEV (MOI=0.1) for 24 h. The mRNA levels of JEV core protein C and LYAR were determined by qRT-PCR. The RNA levels were normalized to 18S rRNA level (mean± S.D. of three independent experiments, *$P < 0.05$ and *** $p < 0.001$, two-tailed Student's t-test).

Table. S1 PCR, RT-PCR primers, small interfering RNAs and FISH probe sequences used in this study.

Table. S2 Host proteins identified by AP-MS. Host proteins and RNP components (PA, PB1, PB2 and NP) co-purified by Flag-PB1. UniProt ID, gene name, peptide sequences and mascot score of identified host and viral proteins in two replicates are listed.

Table. S3 Overlap of host proteins identified between this study and previously published studies. To determine whether any of the genes identified in this study are also identified in previously reported studies, we analyzed the proteins identified by us and those identified in previous six related studies for identifying host proteins interacting with vRNP, RdRp or RNP components. UniProt ID, Gene name, virus binding partners and references are listed. Arabic number 1 represents that the host protein is the binding partner of the indicated viral protein. Cited references are listed in the table.

Table. S4 Gene ontology (GO) analysis and KEGG analysis of the host proteins.

Table. S5 Effect of the host proteins on IAV replication. Pro-viral and anti-viral host factors supported by reported RNAi data from large-scale screens.

Column C: phenotype of host proteins to virus reported by Watanabe et al (2014); Column D: phenotype of host proteins to virus reported by Tipathi et al (2015).

Table. S6 Host-host protein interactions taken from STRING.

Column A: node 1, Column B: node 2, Column C: combined score. Nodes represent proteins, and combined score represents the confidence of the interaction between two proteins.

Figure S1

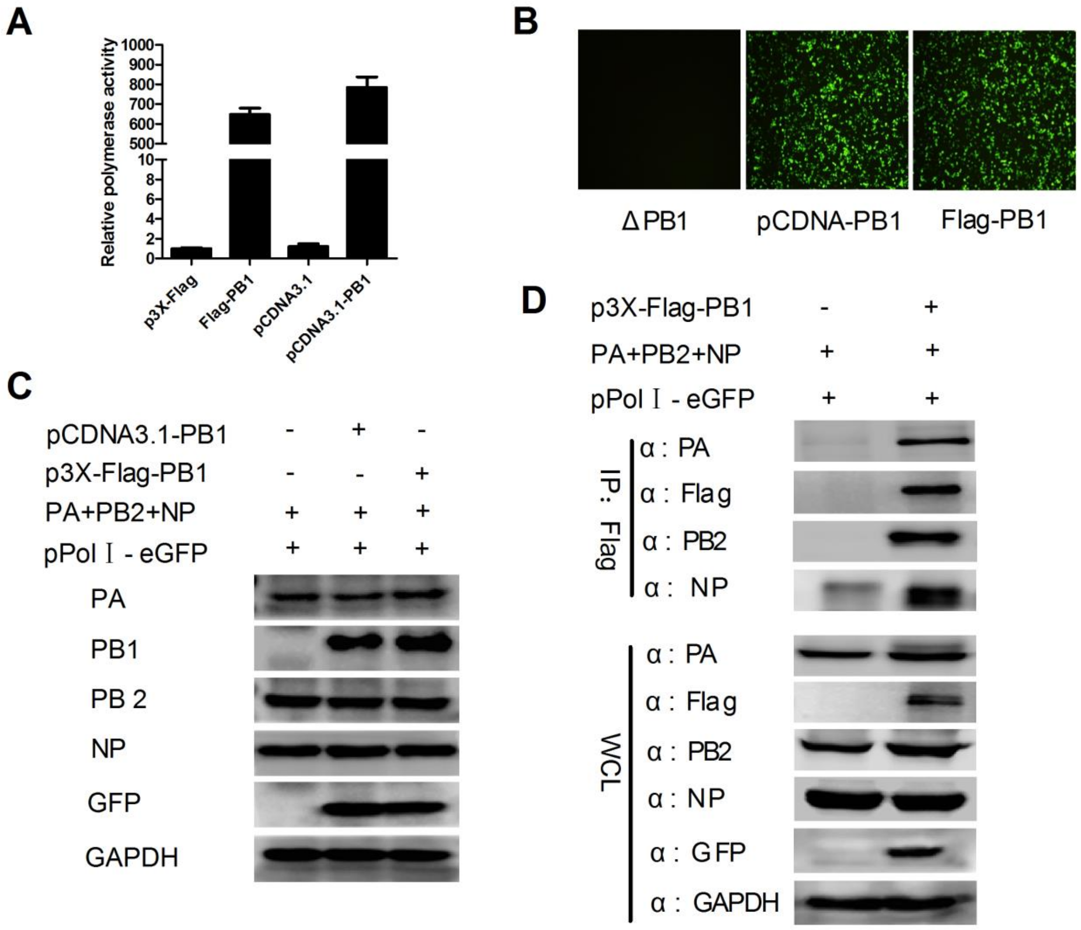
A
Relative polymerase activity
1000
900
800
700
600
500
10
8
6
4
2
0
p3X-Flag
Flag-PB1
pCDNA3.1
pCDNA3.1-PB1
B
ΔPB1
pCDNA-PB1
Flag-PB1
C
pCDNA3.1-PB1 - + -
p3X-Flag-PB1 - - +
PA+PB2+NP + + +
pPolⅠ- eGFP + + +
PA
PB1
PB 2
NP
GFP
GAPDH
D
p3X-Flag-PB1 - +
PA+PB2+NP + +
pPolⅠ- eGFP + +
IP: Flag
α : PA
α : Flag
α : PB2
α : NP
WCL
α : PA
α : Flag
α : PB2
α : NP
α : GFP
α : GAPDH

Figure S2

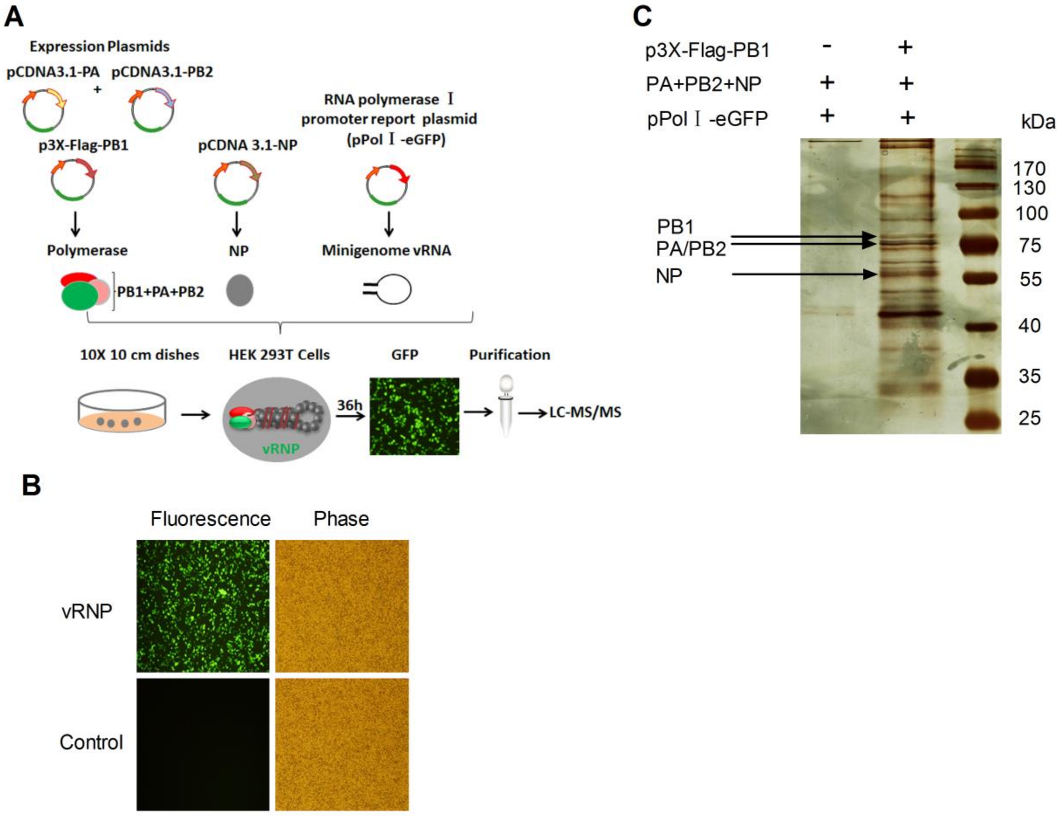
A
Expression Plasmids
pCDNA3.1-PA
pCDNA3.1-PB2
p3X-Flag-PB1
pCDNA 3.1-NP
RNA polymerase Ⅰ promoter report plasmid (pPol Ⅰ -eGFP)
Polymerase
NP
Minigenome vRNA
PB1+PA+PB2
10X 10 cm dishes
HEK 293T Cells
GFP
Purification
vRNP
36h
LC-MS/MS
B
Fluorescence
Phase
vRNP
Control
C
p3X-Flag-PB1
PA+PB2+NP
pPol Ⅰ -eGFP
kDa
PB1
PA/PB2
NP
170
130
100
75
55
40
35
25

Figure S3

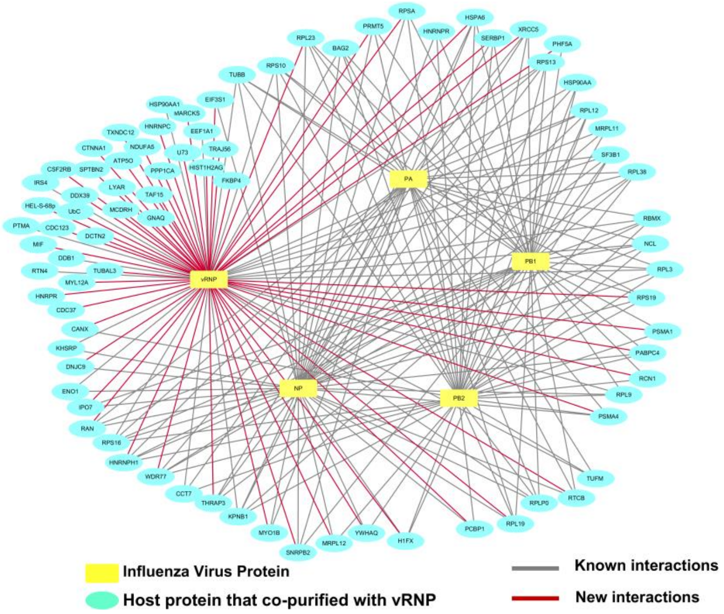
vRNP
PA
PB1
PB2
NP
RPSA
PRMT5
HSPA6
HNRNPR
XRCC5
SERBP1
PHF5A
RPL23
BAG2
RPS13
RPS10
TUBB
HSP90AA
EIF3S1
HSP90AA1
MARCKS
RPL12
MRPL11
TXNDC12
HNRNPC
EEF1A1
CTNNA1
NDUFA5
U73
TRAJ56
SF3B1
ATP5O
CSF2RB
SPTBN2
PPP1CA
HIST1H2AG
RPL38
IRS4
LYAR
FKBP4
DDX39
TAF15
HEL-S-68p
UbC
MCDRH
GNAQ
RBMX
PTMA
CDC123
DCTN2
NCL
MIF
DDB1
RPL3
RTN4
TUBAL3
MYL12A
HNRPR
RPS19
CDC37
CANX
PSMA1
KHSRP
PABPC4
DNJC9
RCN1
ENO1
RPL9
IPO7
PSMA4
RAN
RPS16
HNRNPH1
TUFM
WDR77
CCT7
RPLP0
RTCB
THRAP3
KPNB1
RPL19
PCBP1
MYO1B
YWHAQ
H1FX
MRPL12
SNRPB2
Influenza Virus Protein
Host protein that co-purified with vRNP
Known interactions
New interactions

Figure S4

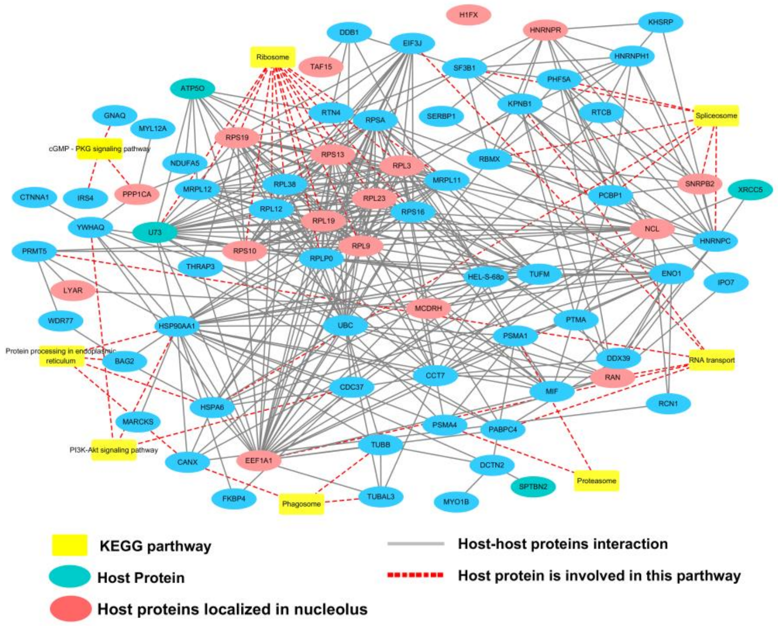
H1FX
HNRNPR
KHSRP
DDB1
EIF3J
Ribosome
TAF15
SF3B1
HNRNPH1
ATP5O
PHF5A
GNAQ
RTN4
RPSA
SERBP1
KPNB1
RTCB
Spliceosome
MYL12A
RPS19
cGMP - PKG signaling pathway
RPS13
NDUFA5
RBMX
RPL3
SNRPB2
XRCC5
CTNNA1
IRS4
PPP1CA
MRPL12
RPL38
RPL23
MRPL11
PCBP1
RPL12
RPS16
YWHAQ
U73
RPL19
NCL
HNRNPC
PRMT5
RPS10
RPL9
RPLP0
THRAP3
HEL-S-68p
TUFM
ENO1
IPO7
LYAR
MCDRH
WDR77
HSP90AA1
UBC
PTMA
PSMA1
Protein processing in endoplasmic reticulum
BAG2
DDX39
RNA transport
RAN
CCT7
CDC37
MIF
RCN1
HSPA6
MARCKS
PSMA4
PABPC4
PI3K-Akt signaling pathway
TUBB
CANX
EEF1A1
DCTN2
SPTBN2
Proteasome
FKBP4
Phagosome
TUBAL3
MYO1B
KEGG parthway
Host Protein
Host proteins localized in nucleolus
Host-host proteins interaction
Host protein is involved in this parthway

Figure S5

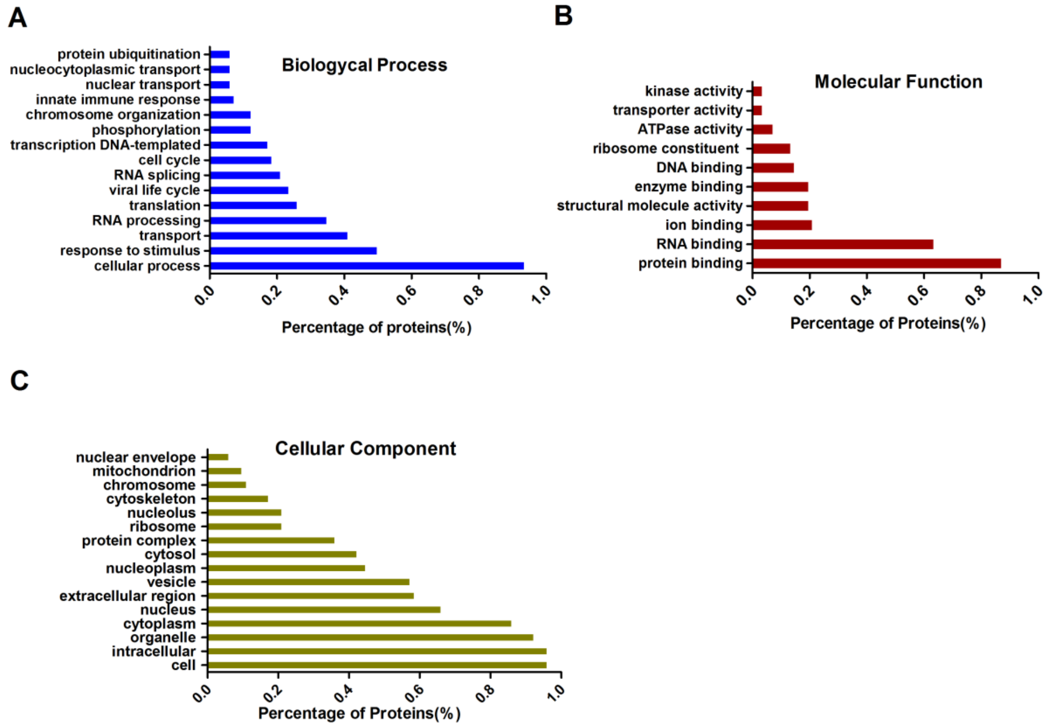
A
Biologycal Process
protein ubiquitination
nucleocytoplasmic transport
nuclear transport
innate immune response
chromosome organization
phosphorylation
transcription DNA-templated
cell cycle
RNA splicing
viral life cycle
translation
RNA processing
transport
response to stimulus
cellular process
0.0
0.2
0.4
0.6
0.8
1.0
Percentage of proteins(%)
B
Molecular Function
kinase activity
transporter activity
ATPase activity
ribosome constituent
DNA binding
enzyme binding
structural molecule activity
ion binding
RNA binding
protein binding
0.0
0.2
0.4
0.6
0.8
1.0
Percentage of Proteins(%)
C
Cellular Component
nuclear envelope
mitochondrion
chromosome
cytoskeleton
nucleolus
ribosome
protein complex
cytosol
nucleoplasm
vesicle
extracellular region
nucleus
cytoplasm
organelle
intracellular
cell
0.0
0.2
0.4
0.6
0.8
1.0
Percentage of Proteins(%)

Figure S6

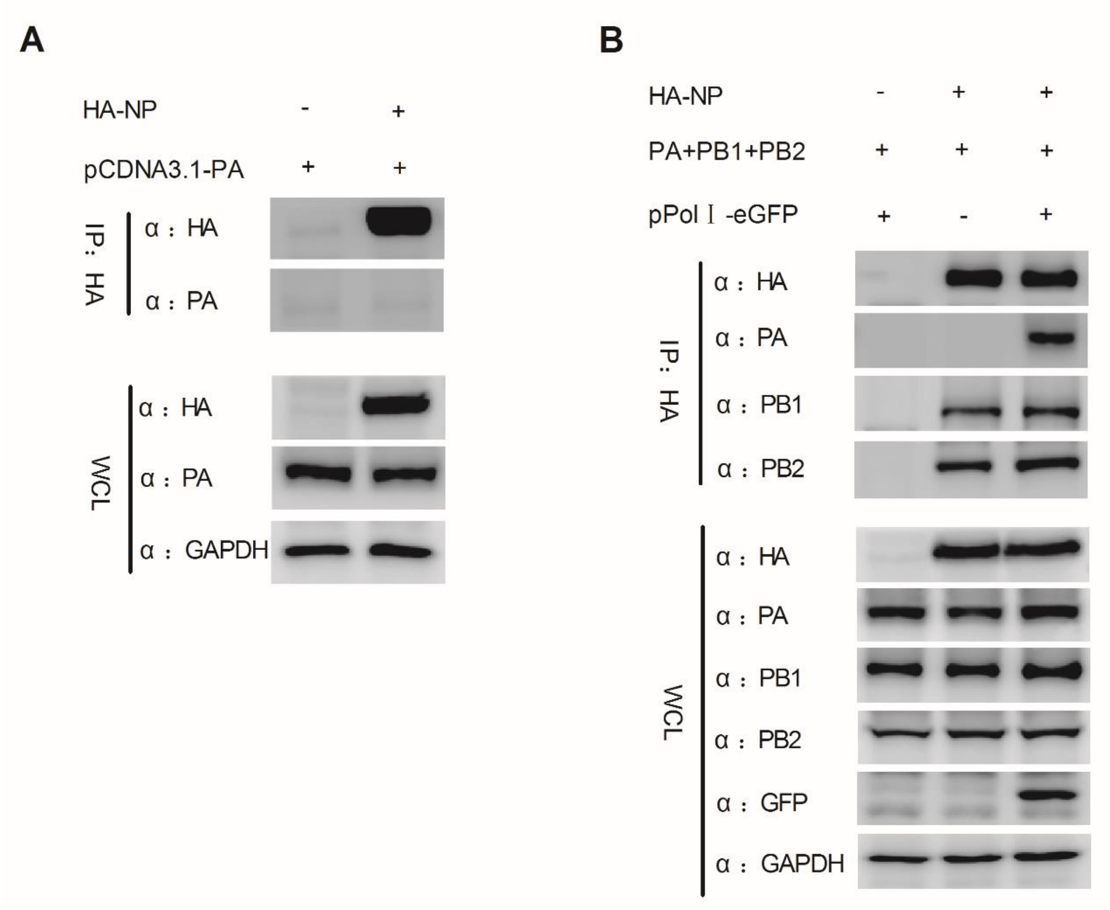
A
HA-NP
pCDNA3.1-PA
IP: HA
α : HA
α : PA
WCL
α : HA
α : PA
α : GAPDH
B
HA-NP
PA+PB1+PB2
pPolⅠ-eGFP
IP: HA
α : HA
α : PA
α : PB1
α : PB2
WCL
α : HA
α : PA
α : PB1
α : PB2
α : GFP
α : GAPDH

280 **Figure S7**

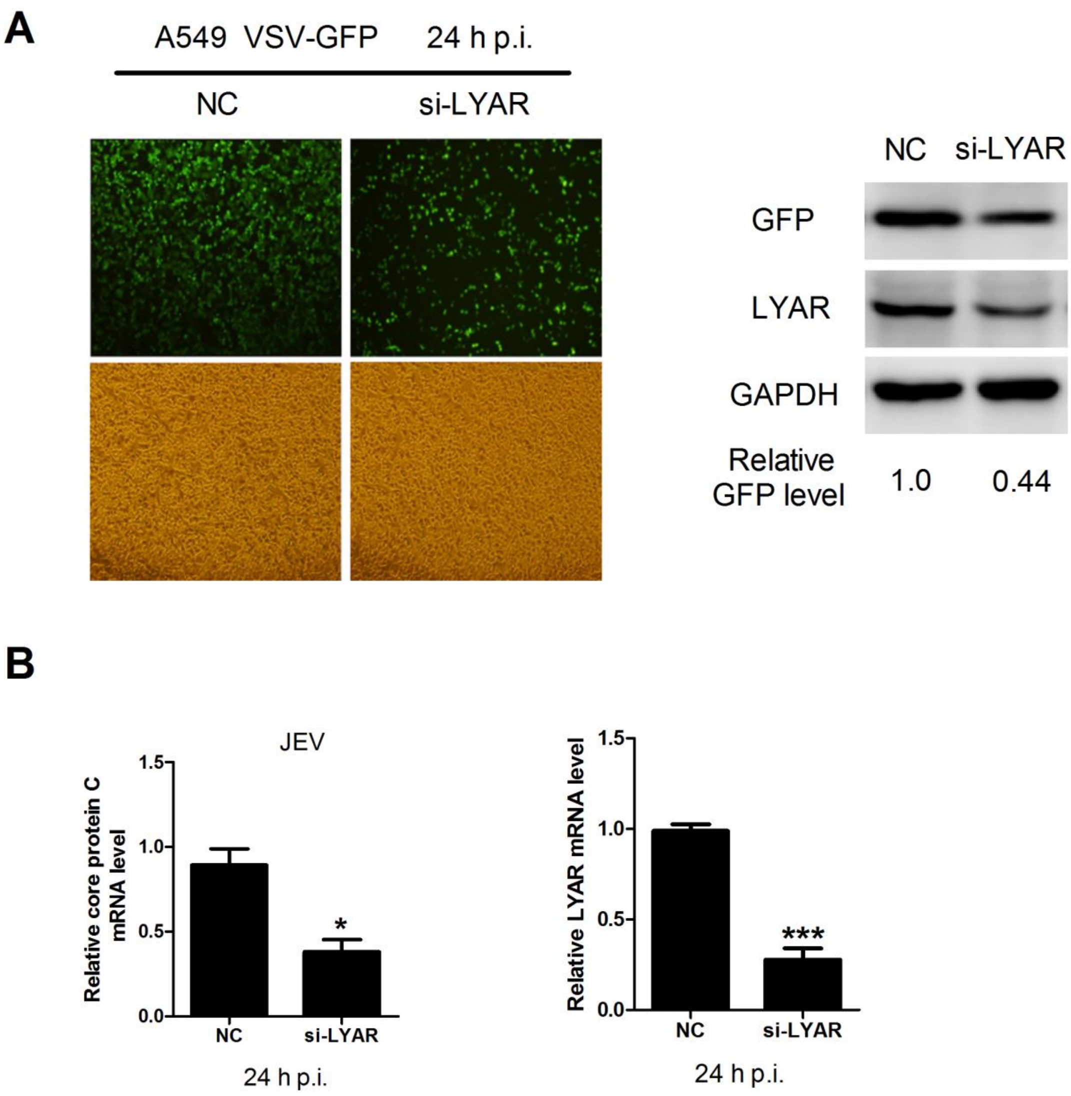

281
